# A Research Hotspot-Guided Meta-Analysis of Anterior Closing-Wedge High Tibial Osteotomy in Revision Anterior Cruciate Ligament Reconstruction

**DOI:** 10.3390/bioengineering13030327

**Published:** 2026-03-12

**Authors:** Xu Liu, Ahmed Abdirahman Ibrahim, Abakar Mahamat Abdramane, Michael Opoku, Pavel Volotovsky, Mikhail Gerasimenko, Yusheng Li, Shiyao Chu, Haitao Long

**Affiliations:** 1 Department of Orthopaedics, Xiangya Hospital, Central South University, 87 Xiangya Road, Changsha 410008, China; 228112308@csu.edu.cn (X.L.); 248119013@csu.edu.cn (A.A.I.); 248119012@csu.edu.cn (A.M.A.); michael_opoku@csu.edu.cn (M.O.); liyusheng@csu.edu.cn (Y.L.); 2 Xiangya School of Medicine, Central South University, Changsha 410083, China; 3 Republican Scientific and Practical Center of Traumatology and Orthopedics, 220024 Minsk, Belarus; volotovski@gmail.com (P.V.); gerasimenko@tut.by (M.G.); 4 National Clinical Research Center for Geriatric Disorders, Xiangya Hospital, Central South University, Changsha 410008, China; 5 Department of Traumatology and Orthopedics, Belarusian State Medical University, 220024 Minsk, Belarus

**Keywords:** anterior cruciate ligament reconstruction, revision, research hotspots, posterior tibial slope, meta-analysis, bibliometric analysis

## Abstract

Background: Revision anterior cruciate ligament reconstruction (ACLR) presents an increasing clinical challenge with higher failure rates than primary reconstruction. However, the evolving research landscape and clinical evidence regarding key biomechanical risk factors remain incompletely synthesized. Methods: A sequential dual methodological approach was applied. First, a bibliometric analysis of the Web of Science Core Collection was performed to map global research trends and identify emerging hotspots. Based on the identified hotspot, a PRISMA-compliant meta-analysis of studies retrieved from PubMed, Embase, the Cochrane Library, and Web of Science was subsequently conducted. Clinical and radiographic outcomes were synthesized using random-effects models in R. Results: The bibliometric analysis included 4213 publications and demonstrated exponential growth in revision ACLR research, identifying posterior tibial slope (PTS) as the dominant research hotspot. A meta-analysis of 11 studies involving 299 patients showed significant postoperative improvements in patient-reported outcomes and objective knee stability measures, along with a mean PTS reduction of 8.72° (95% CI 7.84–9.60; *p* < 0.001), while no significant change in patellar height was observed. The pooled return-to-sport rate was 74% (95% CI 64–82%), and the most common complications were symptomatic hardware and postoperative recurvatum. Conclusions: PTS has emerged as a key focus in revision ACLR research, and addressing this biomechanical factor may be associated with improved functional and radiographic outcomes. However, current evidence is mainly derived from retrospective studies, and further prospective research is needed to confirm long-term efficacy and refine surgical indications.

## 1. Introduction

Revision anterior cruciate ligament reconstruction (ACLR) has emerged as a distinct and increasingly prevalent subspecialty within sports medicine, now constituting 4.1–13.3% of all ACL procedures [1,2]. This prominence is driven by the substantial volume of primary ACLR and their associated failure rates, which can exceed 10% and rise to 34.2% in high-risk cohorts such as younger athletes [3,4]. The clinical significance of revision surgery is underscored by its technical complexity and historically inferior patient outcomes compared to primary reconstruction. These outcomes include higher graft re-rupture rates—reported as high as 20% in a systematic review [5]—and lower, more unpredictable rates of return to sport, with systematic reviews indicating a wide success range of only 13% to 69% [5,6,7]. A comprehensive understanding of failure etiology is therefore paramount. The Multicenter ACL Revision Study Group has categorized the primary causes as traumatic (32%), technical (24%), biologic (7%), or, most commonly, a combination of factors (37%) [8]. Consequently, modern surgical strategies extend beyond simple graft replacement to address technical errors, concomitant meniscal and chondral pathology, and patient-specific anatomical risk factors that alter knee biomechanics and predispose the graft to failure [9,10,11,12,13,14,15].

The complex and challenging nature of revision ACL surgery necessitates a sophisticated approach to evidence synthesis. This need is heightened by the current research landscape: despite a rapidly expanding body of literature, the field remains characterized by heterogeneous studies and a scarcity of high-quality evidence [16,17,18]. To address the discrepancy between research volume and clinical guidance, this study integrates two complementary methodologies. Bibliometric analysis enables the identification of research trends and emerging hotspots within a scientific field [19,20,21]. Complementarily, meta-analysis provides a robust statistical framework for quantitatively synthesizing treatment outcomes across clinical studies [22,23].

The objective of this study is to comprehensively summarize the current research landscape and trends in the field of revision ACLR, with a primary focus on identifying emerging research hotspots. This study hypothesizes that current research hotspots predominantly involve modified surgical techniques aimed at addressing risk factors for failure in revision ACLR. To evaluate the clinical efficacy of these modified techniques, a subsequent meta-analysis is conducted for empirical verification. By integrating bibliometric analysis with meta-analytical approaches, this study aims to provide a more systematic and comprehensive understanding of this research domain.

## 2. Materials and Methods

The meta-analysis was conducted in accordance with the Preferred Reporting Items for Systematic Reviews and Meta-Analyses (PRISMA) and Assessing the Methodological Quality of Systematic Reviews (AMSTAR) guidelines [24,25], and was prospectively registered in the PROSPERO (Registration ID: CRD420251121425).

### 2.1. Bibliometric Analysis

#### 2.1.1. Data Source and Search Strategy

The WOSCC has been suggested by researchers as a reliable source for conducting bibliometric analyses. It is regarded as a highly authoritative database, offering key information including author identities, countries of origin, and publishing journals. Furthermore, WOSCC offers data files formatted to satisfy the specifications of bibliometric tools, including VOSviewer, CiteSpace, and R software. Therefore, we conducted a literature search on 17 April 2025 using WOSCC. The search terms were “revision OR revise OR re-rupture OR failure” AND “anterior cruciate ligament reconstruction OR ACLR OR ACL reconstruction”; the detailed search strategy is illustrated in Table S1. To minimize discrepancies resulting from database updates, all operations were finalized on the same day (17 April 2025).

#### 2.1.2. Data Extraction, Visualization, and Statistical Analysis

Two independent reviewers systematically collected information on publication year, authorship, country of origin, affiliated institutions, keywords, and other related characteristics during the screening process and data extraction. A third reviewer was consulted in the event of disagreement. For publication trend analysis, the bibliometrix package in R (version 4.5.1; R Foundation for Statistical Computing, Vienna, Austria) was used to extract publication data, followed by plotting and fitting analysis using OriginPro 2025 software (OriginLab Corporation, Northampton, MA, USA). Turning point analysis of publication trends was conducted using Joinpoint software (version 5.4.0; National Cancer Institute of the United States). For visualization and bibliometric mapping, VOSviewer (version 1.6.18; Centre for Science and Technology Studies, Leiden University) was employed for co-authorship and clustering analyses, while CiteSpace (version 6.2.4.0; Drexel University, Philadelphia, PA, USA) was used for citation burst analysis. The geographical distribution map was generated using VOSviewer in combination with Scimago Graphica (version 1.0.16; SCImago Research Group, Granada, Spain). R (version 4.5.1) was used to produce stacked area charts. GraphPad Prism (version 9.5.0; GraphPad Software, San Diego, CA, USA) was applied to create donut charts, and additional visualizations were generated, including subgroup analyses of keywords, with trends in the publication of hot keywords and a country-level analysis. The Chord diagram of international collaborations among countries was generated using Charticulator (Microsoft Research, Redmond, WA, USA). The bibliometrix package in R was specifically applied to generate the author analysis plot (Top 10 authors’ publication and citation trends). Microsoft Excel 2019 (Microsoft Corporation, Redmond, WA, USA) was used for manual verification of frequencies and percentages to ensure accuracy.

### 2.2. Meta-Analysis

#### 2.2.1. Literature Search

Based on the preliminary bibliometric analysis, research hotspots in the field of revision ACLR were identified through keyword co-occurrence clustering and hotspot evolution trend analysis. The results indicated that the identified hotspot focused on a specific modified surgical technique in revision ACLR. Accordingly, this hotspot was used as the core research direction to develop and refine a systematic literature search strategy.

This hotspot-driven approach was adopted to integrate bibliometric mapping with quantitative evidence synthesis. Bibliometric analysis enables the identification of emerging research fronts and highly concentrated topics within a research field, thereby providing an objective basis for defining a focused meta-analysis question. By using keyword co-occurrence clustering to identify the most intensively studied surgical modification, the present study aimed to ensure that the subsequent meta-analysis addressed a clinically relevant and well-defined research direction. However, it should be acknowledged that selecting a research topic based on bibliometric hotspots may introduce potential selection bias and therefore requires careful interpretation of the synthesized evidence.

The search strategy combined free-text terms and Medical Subject Headings (MeSH). The core search terms were ((anterior cruciate ligament reconstruction OR ACLR OR ACL reconstruction) AND (revision OR revise OR re-rupture OR failure)) AND (the hotspot). To ensure comprehensive coverage, the electronic search was supplemented with a manual review of reference lists from included articles and relevant systematic reviews.

#### 2.2.2. Study Eligibility

Considering the integrated bibliometric and meta-analysis approach of this study, the inclusion and exclusion criteria for the meta-analysis were established based on the research hotspots identified through keyword analysis. The final selection criteria and characteristics of the included studies are reported in the results section.

#### 2.2.3. Data Extraction

Data extraction from the included studies was independently performed by two authors using a structured information table. Any inconsistencies were initially addressed through discussion, and if consensus could not be achieved, the decision was referred to a third author. Data extraction included the following elements: (1) basic characteristics of the literature: title, first author, year of publication, and country; (2) experimental information: study design, level of evidence, and inclusion and exclusion criteria; (3) patient information: number of patients in each group, type of modified surgical technique applied, gender ratio, age, fixation method, and follow-up; (4) clinical outcomes and radiographic outcome measures.

#### 2.2.4. Quality Assessment

The methodological quality of the included studies was assessed according to study design. For randomized controlled trials (RCTs), the Cochrane Risk of Bias 2 (RoB 2) tool was used [26], which evaluates the following domains: (1) randomization process, (2) deviations from intended interventions, (3) missing outcome data, (4) measurement of the outcome, and (5) selection of the reported result.

For non-randomized studies, the Methodological Index for Non-Randomized Studies (MINORS) instrument was applied [27]. The MINORS tool consists of eight items: (1) a clearly stated aim, (2) inclusion of consecutive patients, (3) prospective data collection, (4) endpoints appropriate to the aim of the study, (5) unbiased assessment of endpoints, (6) an adequate follow-up period, (7) loss to follow-up less than 5%, and (8) prospective calculation of sample size. Each item is scored as 0 (not reported), 1 (reported but inadequate), or 2 (reported and adequate), with a maximum total score of 16. Overall quality is interpreted as 13–16 (high), 9–12 (moderate), and 0–8 (low).

Two reviewers independently assessed the methodological quality of each included study. Any discrepancies were resolved through discussion or consultation with a third reviewer.

#### 2.2.5. Statistical Analysis

All statistical analyses were conducted using the meta package in R (version 4.1.3). For continuous outcomes, data were synthesized using means and standard deviations, or medians and quartiles. The inverse variance method was applied for pooling, and results are presented as mean differences (MDs) with a 95% confidence interval (CI). For dichotomous outcomes, data including the number of events and total patient counts were extracted, and the Mantel–Haenszel method was used for data synthesis. Pooled effect sizes are presented in the form of proportion with a 95% CI. A *p* value less than 0.05 was considered statistically significant. The I^2^ statistic was used to assess heterogeneity between the studies. According to Cochrane’s handbook, a fixed-effects model was applied when the I^2^ value was less than 50%; otherwise, a random-effects model was deemed appropriate. Subgroup analysis was performed if sufficient evidence was available. We also used funnel plots to assess the publication bias for outcomes included in more than ten studies.

## 3. Results

### 3.1. Results of Bibliometric Analysis

#### 3.1.1. Overall Analysis of the Current State of Research

**Figure 1:**
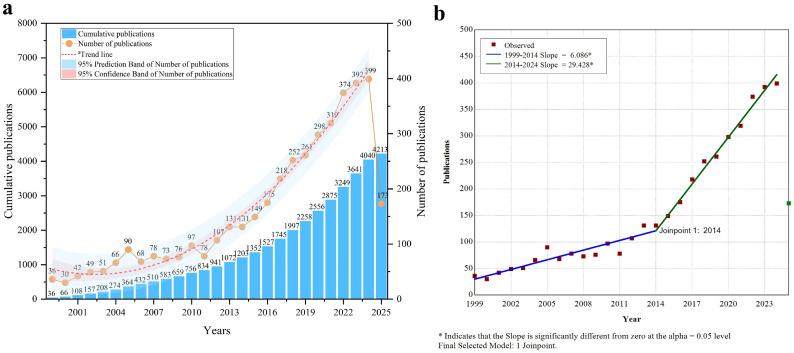
Distribution and trends in ACLR revision surgery research. (**a**) Publication output distribution and trends over time. The red dashed line represents the trend line (y = 60.66231 − 7.00739x + 0.7938x^2^). (**b**) Phases of publication output in ACLR revision surgery research. The asterisk indicates that the slope is significantly different from zero at the α = 0.05 level. Final selected model: 1 joinpoint. The green bar represents the number of publications in 2025, which was not included in the Joinpoint regression analysis.

Productivity analysis in ACLR revision surgery research helps understand the dynamics and emerging trends in the field. Since 1999, we have extracted 4213 publications from the WOSCC database. In 2012, the annual publication volume surpassed 100 for the first time, reaching a peak of 399 publications in 2024. The red dashed line in Figure 1a represents the fitted publication trend line, showing an exponential upward trajectory (y = 60.66231 − 7.00739x + 0.7938x^2^), with an R^2^ value of 0.98022, indicating a good model fit. It should be noted that, as of the date of our retrieval, 2025 has not yet concluded, and therefore, the data is incomplete and unsuitable for fully representing the publication trend. Therefore, the publication data for 2025 was excluded from both the fitting analysis and the turning point analysis. The steady growth trend was further confirmed through node analysis, with 2014 identified as a significant turning point. During the 1999–2014 period, the annual publication slope was 6.086 (slope1), whereas during the 2014–2024 period (slope2), the slope increased to 29.428, indicating a significant rise in publications in the past decade (Figure 1b). A total of 1072 publications (25.4%) were published before 2014, while 3141 publications (74.6%) have been published since 2014.

Analysis of national research output and collaboration patterns helps understand the global progress, inter-country research differences, and emerging collaboration trends in ACLR revision surgery. A total of 78 countries have contributed to the publication of the relevant literature. Figure S1a presents the area stacked chart of annual publication volumes for the top 10 countries, showing a steady increase in the number of publications for these countries. The United States is the leading country in publication volume (*n* = 1575), followed by China (*n* = 330), Germany (*n* = 326), and France (*n* = 219). Notably, China, the second-largest contributor, saw a significant increase in publications around 2014, reaching a peak in the last two years, rising to second place. Through constructing a chord diagram of the top 30 producing countries (Figure S1b), we found that the United States plays a leading role in most international collaborations, maintaining extensive ties with multiple countries. Notably, countries with high publication volumes, such as the United States, China, Germany, France, Italy, and Japan, have close collaborations and strong partnerships. Figure S1c presents the world geographical distribution map of cooperation clusters among countries with more than five publications. Currently, the ACLR revision surgery research field is primarily concentrated in two major research regions: North America and Europe. In contrast, the publication volume in Africa and South America is much lower, with only two and three countries, respectively, surpassing five publications. Figure S2a presents a citation burst analysis, showing that countries like Turkey, Egypt, Vietnam, and India have shown increased activity in this field in recent years and are expected to become new contributors.

Conducting institutional output and collaboration analysis helps analyze the structure of the research field. A total of 3611 institutions have contributed to ACLR revision surgery publications, with the University of Pittsburgh being the leading institution, publishing 258 articles. Additionally, six institutions, including Special Surgery Hospital, Mayo Clinic, The Ohio State University, McMaster University, and Rush University, each published more than 100 articles. The area stacked chart shows that the top 10 institutions have almost all experienced two publication peaks in 2020 and 2023. Notably, McMaster University began publishing a significant number of papers in this field, starting in 2017, and successfully entered the top 10 institutions in recent years (Figure S3a). Institutional categorization shows that hospitals account for nearly half of the publication volume (46.91%), followed by universities (40.91%), research institutions (6.92%), and government agencies (5.26%) (Figure S3b). Figure S3c depicts the collaboration network between major institutions, revealing extensive cooperation among key institutions, with the University of Pittsburgh, Special Surgery Hospital, Mayo Clinic, and The Ohio State University as central nodes in the network. Figure S2b shows the citation burst analysis for institutions, indicating that institutions like Santy Orthopedic Center and FIFA Medical Excellence Center have been the most active since 2020, and these institutions are expected to bring new significant contributions to the ACLR revision surgery field in the future.

Author analysis reveals key information about core authors and collaboration patterns within the academic community. A total of 12,512 authors have contributed to the research in this field, with an average of 6.23 collaborators per study, and only 46 papers were completed by a single author. Figure S4a shows the number of publications and citations for the top 10 authors, indicating a significant increase in both publication volume and citations since 2010, reflecting the rapid development of ACLR revision surgery research. A collaboration network for authors with more than 20 publications was generated using VOSviewer (Figure S4b), showing a co-occurrence network of core authors with eight relatively independent clusters. Within each cluster, collaboration is tight, while there is also extensive collaboration between clusters. In terms of collaboration timing, author collaboration clusters began forming after 2016 and were mostly established by around 2020 (Figure S4c). Specifically, early collaborations (2016–2020) were primarily concentrated within the red and orange clusters, while recent collaborations (2020–2022 and beyond) have been concentrated between the yellow, green, dark blue, and light blue clusters.

#### 3.1.2. Keyword Analysis and Identification of Hotspots in Revision ACLR

**Figure 2:**
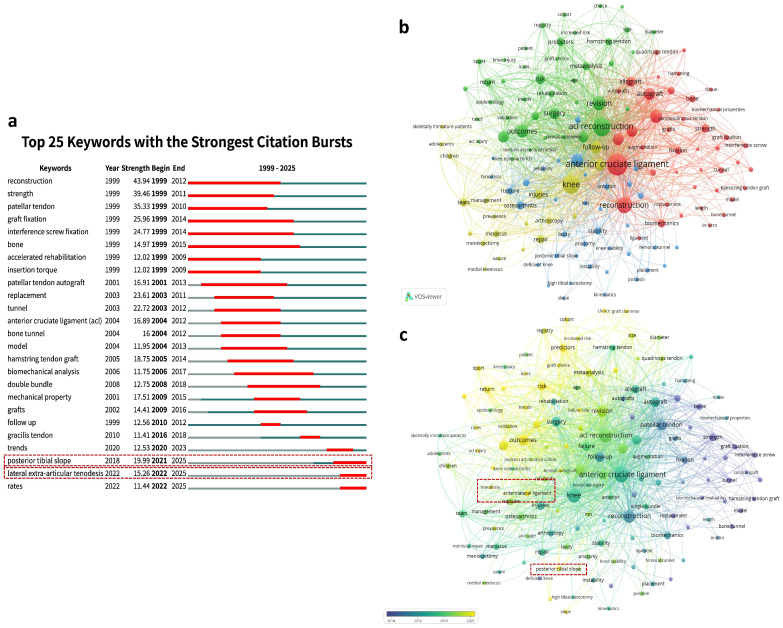
Keyword burst detection and co-occurrence analysis in ACLR revision surgery research. (**a**) Top 25 keywords with the strongest citation bursts between 1999 and 2025, with red segments indicating the periods of intense citation activity. The red segments indicate the citation burst periods of keywords. The blue-green segments represent the period during which the keyword appeared in the dataset, while the gray line indicates the entire study period. (**b**) Keyword co-occurrence network generated by VOSviewer (*n* ≥ 20). (**c**) Temporal overlay visualization of keyword co-occurrence. Each node represents a keyword, with node size proportional to occurrence frequency. Colors denote clusters of related terms, and the thickness of connecting lines reflects the strength of co-occurrence links. Node colors represent the average publication year, ranging from blue (earlier years) to yellow (more recent years).

A total of 7021 keywords were identified through keyword analysis, of which 4654 were author keywords. Citation burst analysis of keywords shows that “posterior tibial slope (PTS)”, and “lateral extra-articular tenodesis (LET)” have become the most frequently occurring keywords since 2022 (Figure 2a). Using VOSviewer, a co-occurrence analysis of keywords occurring more than 50 times identified four distinct research clusters, each representing a major research theme (Figure 2b). Specifically, the green cluster focuses on the clinical outcomes, patient recovery process, and failure risk assessment of ACLR revision surgery, including keywords such as “outcomes”, “failure”, “risk”, “graft rupture”, and “predictors”. This cluster reflects the growing emphasis on identifying prognostic factors and optimizing postoperative management to reduce revision failure. The red cluster addresses surgical techniques, including graft selection and fixation methods, with keywords like “autograft”, “patellar tendon”, “hamstring”, “graft fixation”, and “biomechanics”. These studies primarily explore surgical optimization strategies aimed at improving graft durability and biomechanical performance. The blue cluster focuses on knee stability, kinematics, and anatomy, with representative keywords including “anatomy”, “stability”, “kinematics”, “PTS”, “LET”, and “tunnel placement”. This cluster highlights the biomechanical determinants of knee stability, which are increasingly recognized as critical factors influencing graft survival and revision outcomes. The yellow cluster focuses on meniscal injuries, tears, and meniscectomy, particularly in the treatment of pediatric patients, with keywords including “children”, “adolescents”, “skeletally immature patients”, “meniscus”, and “meniscectomy”. These studies emphasize the unique challenges in skeletally immature patients, where treatment strategies must balance knee stability with preservation of physeal growth.

Further temporal overlay analysis shows the evolution of each cluster and the shifting research hotspots (Figure 2c). According to the temporal analysis, the focus of ACLR revision surgery research has gradually shifted in different directions. The keywords of the red cluster mainly appeared around 2014, focusing on the fundamental techniques of ACLR revision surgery, such as graft types and fixation methods, aiming to optimize surgical techniques and improve the success rate. By 2018, the research focus shifted towards the blue and yellow clusters. The blue cluster discusses knee stability, biomechanics, and bone tunnel distribution, particularly focusing on how refined surgical techniques and accurate positioning can enhance postoperative knee stability and functional recovery. Meanwhile, the yellow cluster focuses on the treatment of pediatric patients and meniscal injury repair, addressing unique challenges and emphasizing personalized treatment plans based on the skeletal development of young patients. After 2020, the green cluster became the primary focus, with research concentrating on the long-term effects of surgery and the analysis of risk factors for surgical failure, aiming to assess the long-term outcomes, complication management, and failure risks of ACLR revision surgery. Research at this time increasingly focuses on how to improve the long-term success rate of surgery, reduce failure risks, and provide more personalized treatment and long-term follow-up care for patients. Overall, research on ACLR revision surgery has shifted from initial technical improvements to postoperative stability, long-term outcomes, and failure risk analysis, with an increasing focus on personalized treatment for specific patient groups, reflecting the deepening and refinement of research in this field.

Notably, in the blue cluster, the keywords “posterior tibial slope” and “lateral extra-articular tenodesis” are shown in yellow, indicating that they are recent discussion hotspots, consistent with previous citation burst analysis results. Both of these keywords directly relate to knee biomechanical stability and postoperative functional recovery, which have been widely discussed in recent biomechanical and clinical studies [12,28]. Both of these keywords directly relate to knee biomechanical stability and postoperative functional recovery. Tenodesis helps strengthen knee ligament stability, especially during the critical period after reconstruction, while posterior tibial slope is a crucial factor affecting knee kinematics and stability. Lowering the slope through tibial osteotomy helps enhance knee stability, thereby reducing the risk of surgical failure. Further analysis of the publication trends for these two research directions in the 4213 retrieved publications was conducted, along with an analysis of the publication volumes from the top 10 countries. The data shows a significant increase in the annual publication volume for both research directions, further validating their emerging significance in ACLR revision surgery research (Figure S5a). The United States leads both research directions of lateral extra-articular tenodesis and posterior tibial slope, highlighting its dominant position in ACLR revision surgery research. Specifically, the U.S. has significantly more publications than other countries in the field of posterior tibial slope, demonstrating a high concentration of research power in this area (Figure S5b,c). Additionally, Italy, France, and Germany rank among the top five in both research directions, demonstrating their strong research presence in ACLR revision surgery. China tends to focus more on posterior tibial slope research, although its publication volume still lags behind the U.S. and some European countries (Figure S5c).

Through keyword analysis, we have identified the current research hotspots in ACLR revision surgery as “posterior tibial slope” and “lateral extra-articular tenodesis”, while systematic reviews and meta-analyses have validated the efficacy of lateral extra-articular tenodesis [29]. There is still a gap in meta-analyses regarding tibial osteotomy combined with ACLR revision surgery to reduce posterior tibial slope. To further evaluate the efficacy of this procedure, we conducted a meta-analysis.

### 3.2. Results of Meta-Analysis

#### 3.2.1. Inclusion, Exclusion, and Study Selection Criteria

##### Inclusion and Exclusion Criteria

Studies were included if they met the following criteria: (1) exclusively included cases with prior ACL reconstruction failure; (2) confirmed pathological posterior tibial slope (≥12°); (3) involved patients undergoing an anterior closing-wedge high tibial osteotomy (ACW-HTO) as an adjunct to revision ACL reconstruction; (4) were published in English and Chinese language; (5) involved human studies; (6) had a publication date from inception to 31 May 2025; (7) reported a minimum follow-up of six months; and (8) represented randomized controlled trials (RCTs), prospective studies, or retrospective analyses with Oxford Centre for Evidence-Based Medicine (CEBM) 2011 Levels of Evidence (LoEs) 1–4.

Exclusion criteria comprised: (1) studies involving patients with normal PTS (<12°); (2) multi-ligament knee injuries; (3) severe osteoarthritis on radiographic evaluation; (4) primary ACLR failures attributed to technical errors (e.g., malpositioned tibial or femoral tunnels); (5) cases requiring combined coronal and sagittal tibial osteotomies; (6) studies utilizing surgical techniques other than ACW-HTO; (7) biochemical or in vitro studies; (8) case reports, preclinical studies, editorials, book chapters, technical reports, and review articles.

##### Literature Selection

**Figure 3:**
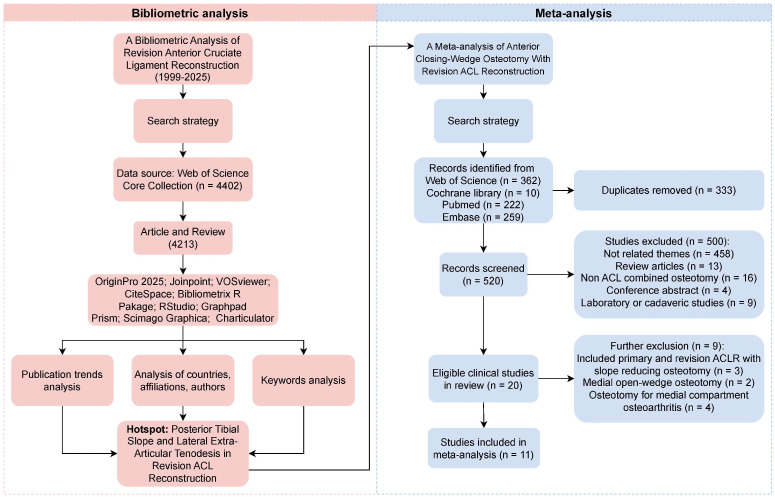
Overall workflow of the study. A schematic diagram illustrating the overall workflow of the study, including the bibliometric analysis and meta-analysis processes.

A comprehensive systematic search was conducted across four electronic databases, PubMed (*n* = 222), Embase (*n* = 259), Web of Science (*n* = 362), and the Cochrane Library (*n* = 10), yielding 853 total records. After removing 333 duplicates, 520 unique studies underwent title and abstract screening. This process identified 20 articles for full-text evaluation. Nine studies were excluded based on predefined criteria: studies included primary and revision ACLR with slope-reducing osteotomy (*n* = 3), medial open-wedge osteotomy (*n* = 2), and medial compartment osteoarthritis (*n* = 4). The final inclusion consisted of 11 eligible studies [30,31,32,33,34,35,36,37,38,39,40], 10 of which were retrospective case series and one prospective case series. The study selection process is detailed in Figure 3.

#### 3.2.2. Basic Characteristics of the Literature

The 11 included studies, comprising 10 retrospective case series and one prospective case series, involved 299 patients. The studies were published in English and Chinese-language journals between 2014 and 2025. The sample size ranged from 5 to 64 patients, with a mean age of 24 to 34 years. The proportion of male patients was 198 (66.2%), and that of females was 101 (33.8%). The mean follow-up of the patients was 6 to 60 months. All studies included ACLR failure with high PTS as an indication for surgery, and most studies used a PTS > 12° as the indication for a slope-reducing osteotomy. The basic characteristics of the included studies are shown in Table S1.

In a pooled analysis of 299 patients from 11 studies, all individuals underwent a second revision ACL surgery with an ACW-HTO. This combined procedure was performed in a single stage for 55.6% of patients and in a two-stage manner for 44.4% of patients. The surgical approach for the ACW-HTO varied, with the trans-tuberosity technique being most common (39.5%), followed by supra-tuberosity (34.4%) and infra-tuberosity (26.1%) approaches. The LET was performed in addition to ACLR and ACW-HTO in 35.8% (*n* = 107) of patients. Surgical techniques, procedure staging (single or two-stage), graft types, and associated meniscal procedures are detailed in Table S2.

#### 3.2.3. Results of Quality Assessment

The methodological quality of the included case series and non-randomized studies was evaluated using the modified MINORS criteria, yielding scores between 8 and 12. While most studies exhibited clearly defined aims and consistent inclusion of consecutive patients, the overall methodological rigor was moderate. Key limitations prevalent across the literature included the frequent absence of prospective data collection, insufficient sample size justification, and inadequate reporting on unbiased endpoint assessment. Only a minority of studies implemented a prospective design or achieved comprehensive follow-up. A detailed presentation of the individual risk of bias for all included studies is provided in Table S3.

#### 3.2.4. Clinical Outcomes

##### IKDC Score

**Figure 4:**
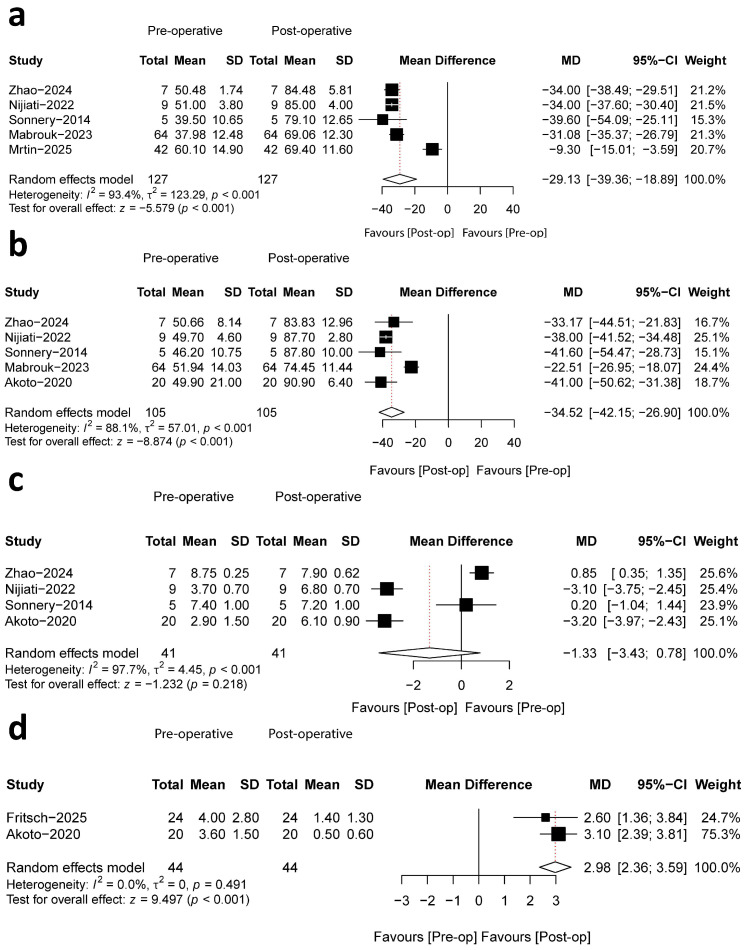
Forest plots illustrating the mean differences for pre- and postoperative IKDC score (**a**), Lysholm score (**b**), Tegner score (**c**), and VAS score (**d**) [30,31,32,33,34,35,36].

Data on the IKDC score, reported in five studies with a collective sample of 127 patients, were analyzed [30,31,32,33,34]. Each study included comparative pre- and postoperative measurements. The reported mean preoperative scores ranged from 37.98 to 60.10. Postoperatively, mean scores demonstrated a substantial increase, ranging from 69.06 to 85.00. The calculated mean improvement ranged from 9.30 to 39.60 points across studies. The meta-analysis synthesizing these findings indicated that the increase in IKDC scores was statistically significant (MD, −29.13; 95%CI, −39.36~−18.89; *p* < 0.001; I^2^ = 93.4%) (Figure 4a).

##### Lysholm Score

The Lysholm score was evaluated across five studies involving a total of 105 patients [30,31,32,33,35]. All studies provided comparative pre- and postoperative data. The collective analysis revealed a statistically significant increase in Lysholm scores postoperatively (MD, −34.52; 95%CI, −42.15~−26.90; *p*< 0.001; I^2^ = 88.1%) (Figure 4b). Preoperative means ranged from 49.70 to 51.94, rising to 74.45 to 90.90 postoperatively, corresponding to a mean improvement of 22.51 to 41.60 points.

##### Tegner Score

Tegner activity scale scores were reported in four studies comprising 41 patients. Pooled meta-analysis of pre- and postoperative data indicated no significant change in activity levels, with similar scores reported across both time points (MD, −1.33; 95%CI, −3.43~0.78; *p* = 0.218; I^2^ = 97.7%) (Figure 4c) [30,31,32,35].

##### VAS Score

Two studies, each with a 2-year follow-up period, reported VAS scores. The meta-analysis of comparative pre- and postoperative results indicated a statistically significant reduction in VAS scores, signifying improved pain outcomes (MD, −2.98; 95%CI, 2.36~3.59; *p* < 0.001; I^2^ = 0.0%) (Figure 4d). The mean preoperative values (range: 3.60–4.00) declined to a postoperative range of 0.40–1.40, representing a mean absolute improvement of 2.60 to 3.10 points [35,36].

#### 3.2.5. Radiographic Outcomes and Physical Examinations

##### PTS

**Figure 5:**
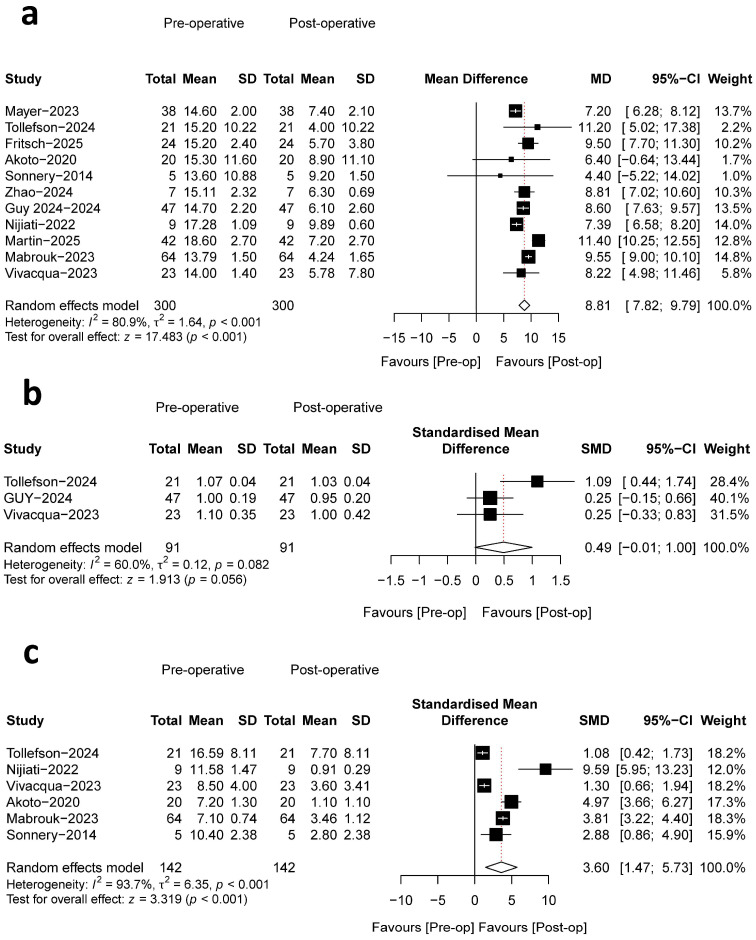
Forest plots illustrating the mean differences between pre- and postoperative radiographic measurements for Posterior Tibial Slope (**a**), patellar height indices (Caton–Deschamps Index and Insall–Salvati Index) (**b**), and anterior tibial translation (**c**) [30,31,32,33,34,35,36,37,38,39,40].

PTS was evaluated across 11 studies, with a collective sample size of 299 patients, all of which provided comparative pre- and postoperative data. The pooled meta-analysis revealed a statistically significant reduction in PTS, with mean values decreasing from a preoperative range of (13.6–18.6°) to a postoperative range of (4.00–9.89°). This corresponded to a mean difference (MD, 8.72°; 95% CI, 7.84–9.60; *p* < 0.001; I^2^ = 80.2%) (Figure 5a), indicating a pronounced effect of the surgical intervention in slope reduction. The methodologies for PTS measurement varied; six studies utilized the medial tibial plateau referenced to the tibial anatomic axis [30,31,32,35,36,40], two employed the lateral tibial plateau [33,38], and a single study applied the mean of both plateaus [37], while two studies did not report their measurement method [34,39].

##### Patellar Height

Patellar height was assessed in the included studies using two standardized radiographic indices: the Caton–Deschamps Index (CDI) and the Insall–Salvati Index (ISI). Three studies reported outcomes utilizing the CDI [37,38,39], while one study employed the ISI [37]. A random-effects meta-analysis was performed to evaluate changes in patella height following the surgical intervention, utilizing combined CDI and ISI measurements. The pooled analysis demonstrated a standardized mean difference (SMD) of 0.25 (95% CI: −0.33 to 0.83) (Figure 5b), indicating a small to moderate increase in patella height postoperatively that did not reach statistical significance (z = 1.913, *p* = 0.056). These negligible changes confirm that the surgical procedure did not significantly alter patellar height and did not induce iatrogenic patella baja in any cohort. Two studies reported no significant difference between pre- and postoperative values [37,38], while one study reported a statistically significant increase in patellar height postoperatively, yet CDI still remained within normal limits (CDI ≤ 1.2) [39]. Substantial heterogeneity was observed among the included studies (I^2^ = 60.0%, τ^2^ = 0.12, *p* = 0.082), suggesting moderate variability in effect sizes.

##### Anterior Tibial Translation (ATT)

Six studies reported pre- and postoperative ATT [31,32,33,35,38,39], which demonstrated a significant reduction in ATT postoperatively. The overall pooled effect was substantial and statistically significant (SMD = 3.60, 95% CI: 1.47 to 5.73, *p* < 0.001) (Figure 5c), indicating a profound improvement in anterior knee stability. Considerable heterogeneity was observed among the studies (I^2^ = 93.7%, *p* < 0.001). This significant heterogeneity is likely attributable to methodological differences in ATT measurement, which utilized five distinct techniques across the studies.

##### Physical Examinations

The pivot-shift test was assessed in four studies [31,32,33,35], while the Lachman test was documented in two studies [35,36]. A consistent improvement in pivot-shift test grades was observed across four studies reporting this outcome. The most pronounced result was a universal conversion from grade 3 preoperatively to grade 0 postoperatively in one study [35]. Other studies reported favorable shifts from higher grades (II–III) to lower grades (0–II). However, one larger study with a heterogeneous preoperative population demonstrated that while most patients improved, 14 patients had persistent moderate (grade II) laxity and 3 patients had persistent severe (grade III) laxity at the final postoperative assessment [33]. A summary of the pivot-shift test is provided in Table S4.

Two studies reported Lachman test outcomes, demonstrating a statistically significant improvement in anterior stability postoperatively. One study documented a firm endpoint in 17 patients postoperatively, with a residual soft endpoint in six and gross laxity in one [36]. A second study quantified this improvement, reporting preoperative pathological laxity in all patients (Grade 2: *n* = 11; Grade 3: *n* = 9) compared to postoperative results where only a single case of Grade 1 laxity was observed (*p* < 0.001). This represents a significant reduction in anterior translational laxity following a combined slope-correction osteotomy and revision ACLR [35]. The detailed grade of the Lachman test is presented in Table S4.

#### 3.2.6. Return to Sports

**Figure 6:**
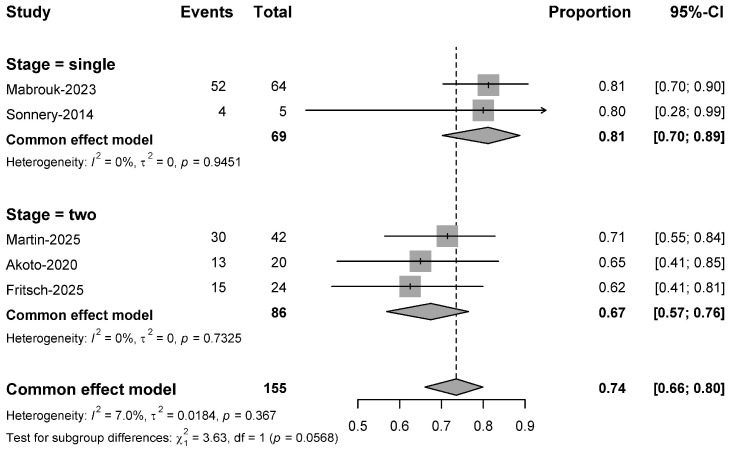
Forest plot for the meta-analysis of RTS rates [32,33,34,35,36].

The pooled analysis demonstrated that 74% of patients (95% CI: 0.64 to 0.82) successfully returned to their pre-injury sport level postoperatively, with low heterogeneity (I^2^ = 9.0%, *p* = 0.355) confirming consistent outcomes across studies [32,33,34,35,36]. Furthermore, a subgroup analysis identified a strong, near-significant trend (*p* = 0.057) indicating that single-stage procedures are associated with higher RTS rates of 81% (95% CI: 0.70 to 0.89) compared to 67% (95% CI: 0.57 to 0.76) for two-stage procedures, pointing to a clinically important effect (Figure 6).

#### 3.2.7. Adverse Events and Complications

The analysis identified a distinct profile of complications. Symptomatic hardware was the most frequently reported issue, with an incidence of 20.0%. Postoperative recurvatum was the second most common complication, occurring in 16.6% of reported cases. Graft failure was observed in 5.3% of cases, while surgical site infection was the least common complication at 2.3%. Other complications reported in the included studies, including hematoma, arthrofibrosis, and complex regional pain syndrome, had a collective incidence of 6.1% (Table S5). These rates are derived specifically from studies that reported each complication, as reporting was not uniform across all publications. The specific breakdown of complication types and their respective incidence rates is detailed in Table S6.

#### 3.2.8. Sensitivity Analysis

Sensitivity analyses were conducted for outcomes with substantial heterogeneity (I^2^ > 50%) and including more than two primary studies, namely IKDC score, Lysholm score, Tegner score, ATT, patellar height Indices, and PTS. Across all assessed outcomes, leave-one-out sensitivity analyses demonstrated that sequential exclusion of any single study did not result in meaningful changes in the pooled effect estimates, confirming the robustness and stability of the primary findings (detailed results are provided in Supplementary Materials S3).

Notably, for certain outcomes, statistical heterogeneity was markedly reduced after omission of specific individual studies, suggesting the presence of influential studies contributing disproportionately to between-study variability. Specifically, heterogeneity was substantially reduced for ATT after omitting Vivacqua 2023 [39], for the IKDC score after omitting Martin 2025 [34], and for the Lysholm score after omitting Mabrouk 2023 [33]. For patellar height indices, heterogeneity remained negligible across all leave-one-out analyses, regardless of which study was excluded. Collectively, these findings indicate that while heterogeneity in some outcomes was partially driven by individual influential studies, the direction, magnitude, and statistical significance of the pooled effects were not dependent on any single study, supporting the validity of the overall conclusions.

For PTS, leave-one-out analyses did not result in a meaningful reduction in heterogeneity, indicating that heterogeneity was not driven by any single study. To further explore this issue, subgroup analyses were conducted according to the anatomical reference used for PTS measurement (medial tibial plateau, lateral tibial plateau, mean of both plateaus, and not reported). These subgroup analyses demonstrated a substantial reduction in heterogeneity within more methodologically homogeneous subgroups, supporting the notion that the observed heterogeneity in PTS was primarily attributable to differences in measurement methodology rather than instability of the pooled effect (Supplementary Materials S3).

#### 3.2.9. Publication Bias

Publication bias was assessed for the PTS outcome. Visual inspection of the funnel plot revealed no potential publication bias for the PTS outcome (Figure S6).

## 4. Discussion

This study employed an integrated bibliometric and meta-analytic framework to map the intellectual structure and evaluate the clinical evidence within revision ACLR research. First, through bibliometric analysis of 4213 publications since 1999, we observed rapid development in revision ACL research, with a notable surge since 2022. The intellectual landscape has been predominantly shaped by North American and European institutions, with the United States leading in output and serving as the central hub for international collaboration. High-productivity institutions like the University of Pittsburgh, Hospital for Special Surgery, and Mayo Clinic form the core of this collaborative network, which is further characterized by tightly knit, influential author clusters that have consolidated since 2016. Bibliometrics, as a research method, has been widely applied to map the intellectual structure of scientific fields. It involves quantitative analysis of the literature to reveal evolving trends and research directions [41,42]. In particular, keyword citation burst analysis is a powerful tool for identifying emerging frontiers by tracking frequently cited terms [43]. Comprehensive keyword analysis identified “PTS” and “LET” as dominant contemporary research hotspots, reflecting the field’s evolution from technical considerations toward addressing underlying biomechanical etiology.

ACLR represents one of the most successful orthopaedic procedures worldwide, consistently demonstrating excellent clinical and functional outcomes in the majority of patients [44]. However, a subset of cases results in failure, with revision ACLR being associated with inferior clinical scores and higher re-failure rates compared to primary reconstruction [45,46]. This has driven extensive research into identifying modifiable risk factors, among which proximal tibial geometry (specifically PTS) has emerged as a critical determinant. A robust body of clinical evidence has established a strong correlation between elevated PTS (>12°) and an increased risk of ACL graft failure, with failure rates escalating proportionally to the degree of slope [40,47,48].

The relationship between PTS and ACL failure is substantiated by foundational biomechanical studies. Bernhardson et al. established that a steeper “PTS > 12°” creates a strong linear increase in ACL graft force under axial load, providing a biomechanical explanation for the elevated clinical failure rates observed in patients with steep slopes [49]. This relationship is further elucidated by the work of Agneskirchner et al., who demonstrated that an increased PTS shifts the tibiofemoral contact point anteriorly, thereby elevating pressure on the anterior tibial plateau and increasing anterior translational forces [50]. Dejour and Bonnin quantified that each 10° increase in PTS results in 6 mm of anterior tibial translation relative to the femur, thereby increasing strain on the ACL graft [51]. Furthermore, a higher PTS amplifies quadriceps-induced anterior drawer forces during knee extension, creating a linear relationship between slope and graft load that predisposes to fatigue failure [49,52,53]. These biomechanical data provide the mechanistic rationale for the clinical observations, confirming that excessive PTS creates a hostile environment for graft longevity and validating the rationale for slope-reducing osteotomy in individuals at risk.

For patients with recurrent ACL graft failure and a PTS > 12°, ACW-HTO is indicated to correct the underlying biomechanical deficit [32,54,55]. This meta-analysis found that the combined ACW-HTO and ACLR procedure consistently produced significant improvements in functional scores (IKDC, Lysholm, Tegner, and VAS) and demonstrated good clinical and radiological outcomes with few complications in the context of ACL revision. The surgical management of excessive PTS via ACW-HTO necessitates careful consideration of the osteotomy’s relationship to the tibial tuberosity, as improper placement can induce iatrogenic patella baja or alta [56,57]. In general, there are three locations for creation of an ACW-HTO relative to the tibial tuberosity: supratuberosity, transtuberosity, and infratuberosity [56]. Based on current clinical experience, the supratuberosity osteotomy avoids patellar tendon detachment but may compromise tibial tunnel positioning [58,59,60]. Alternatively, the transtuberosity approach is more effective at avoiding convergence with the ACL tunnel but requires detachment of the patellar tendon [32,35]. In contrast, the infratuberosity approach preserves the tuberosity; however, its more distal and oblique tibial cut necessitates stronger fixation [61]. In this systematic review, the transtuberosity approach was the most frequently used technique. Furthermore, there were no cases of tibial tuberosity nonunion or delayed union across all ACW-HTO types, and no instances of patella baja or alta were observed, suggesting that all 3 ACW-HTO techniques provide reliable healing potential.

In the evaluation of surgical interventions for young, active individuals, the rate of return to preinjury sporting activity (RTS) serves as a paramount endpoint. This systematic review and meta-analysis of five studies (*n* = 155 patients) found a pooled RTS rate of 74%, which aligns with rates reported after primary ACL reconstruction, where Sepulveda et al. observed an 81% RTS rate [62]. An interesting finding of this systematic review was the consistent trend toward superior RTS outcomes after single-stage procedures compared to two-stage approaches (81% vs. 67%), potentially attributable to the prolonged recovery and psychological burden of two-staged surgeries. However, this advantage must be interpreted with caution due to overlapping confidence intervals and potential selection bias, as more complex cases may be directed toward two-stage protocols [55,63]. Ultimately, the decision to return to sport is multifactorial; as noted in one series by Mabrouk et al., 72.7% of non-returning patients claimed “knee-related issues” encompassing psychological factors and career decisions [33], highlighting that RTS is shaped by a confluence of elements beyond the surgical sequence alone.

This meta-analysis revealed a pooled graft failure rate of 5.3%, which stands in notable contrast to the higher failure rates, often up to 33%, reported for revision ACLR in general [64]. The failure rate for the combined procedure is comparable to, or even better than, the 3–25% range reported for primary ACL reconstruction [64,65]. This collective evidence strongly suggests that slope-reducing osteotomy is an effective adjunct for reducing graft failure risk in revision ACL reconstruction with excessive PTS > 12°. ACW-HTO is a technically demanding procedure associated with potential complications, including general surgical risks such as popliteal neurovascular lesions, tibial tubercle rupture, pseudoarthrosis, increased operative time, and prolonged rehabilitation [32]. A primary biomechanical concern is the risk of iatrogenic genu recurvatum due to the intentional flattening of the PTS, which alters tibial plateau geometry [66,67], potentially leading to an inadvertent knee recurvatum. Notably, in the three studies from this systematic review that reported this finding, all affected patients were asymptomatic [33,35,36]. However, other research indicates that this complication may lead to chronic pain and painful hyperextension of the knee during walking and standing [68,69]. Hardware removal due to pain was required in 25 of 125 (20.0%) of patients. Only studies utilizing plate fixation reported symptomatic hardware issues. Mayer et al. noted irritation at the surgical site, linking it to the anterior positioning of the internal plate fixator after ACW-HTO [40]. Due to this challenging risk profile, many authors reserve ACW-HTO for revision ACLR settings [32,35,70], but it is emphasized that in experienced hands, the procedure is effective and can be performed with a low overall risk of complications [59,61].

This study integrates bibliometric and meta-analytic approaches and is strengthened by its rigorous, data-driven design, which objectively maps the evolution and research hotspots of revision ACLR and provides a focused quantitative synthesis of ACW-HTO in patients with documented ACL reconstruction failure. Notably, this review represents the first dedicated evaluation of ACW-HTO in a clinically homogeneous population with excessive PTS, ensuring a direct and targeted assessment of this technique [71]. These findings highlight the potential clinical importance of addressing biomechanical risk factors such as PTS in revision ACLR and may inform surgical decision making in patients with recurrent ACL insufficiency [39]. Future prospective studies with standardized measurement protocols and longer follow-up are warranted to further clarify the long-term clinical effectiveness and indications for slope-reducing osteotomy [72].

However, several limitations should nonetheless be acknowledged. First, the bibliometric analysis was based solely on the WOSCC and restricted to English-language publications, which may have excluded relevant studies indexed in other databases or published in other languages, thereby limiting the comprehensive representation of global research activity. Additionally, the hotspot-driven design of the meta-analysis, in which the research question was derived from bibliometric keyword clustering, may introduce a degree of selection bias and potential confirmation bias. Although this strategy helps focus the analysis on emerging research fronts, it may also preferentially emphasize topics that are already highly represented in the literature. Second, the systematic review is constrained by the available evidence, which consists predominantly of small, retrospective case series with low methodological quality and without control groups. Third, the absence of a standardized protocol for PTS measurement across studies, including differences in calculation methods, anatomical reference points, and radiographic techniques, introduces a risk of measurement bias and limits comparability of outcomes. Finally, the generally short follow-up durations preclude robust evaluation of long-term durability and clinical effectiveness. These limitations underscore the need for future prospective, controlled studies with standardized measurement protocols to establish more definitive evidence.

## 5. Conclusions

In conclusion, this bibliometric analysis demonstrates that revision ACLR represents a rapidly evolving research field. The findings indicate that PTS has emerged as an important contemporary research focus, reflecting increasing attention to anatomical risk factors associated with graft failure. The subsequent meta-analysis suggests that combining ACW-HTO with revision ACLR may provide favorable clinical outcomes in patients with excessive PTS, including improvements in knee stability, PROs, and RTS, while maintaining an acceptable complication profile. These findings highlight the potential role of slope-reducing osteotomy as a surgical strategy for patients with recurrent ACL insufficiency and increased PTS. However, given that the current evidence is primarily derived from retrospective case series, further prospective controlled studies with standardized measurement protocols and longer follow-up are required to better clarify the long-term clinical effectiveness of this combined procedure.

## Data Availability

The data supporting the findings of this study are derived from publicly available sources and are available from the corresponding author upon reasonable request.

## Supplementary Materials

The following supporting information can be downloaded at https://www.mdpi.com/article/10.3390/bioengineering13030327/s1: Supplementary File S1 presents the detailed search strategies used for the bibliometric analysis and meta-analysis across all databases. Supplementary File S2 contains the supplementary figures (SFigures) supporting the main results, including additional bibliometric visualizations and meta-analytic plots. Supplementary File S3 provides the results of sensitivity analyses conducted to assess the robustness of the pooled estimates. Table S1: Basic characteristics of included studies. Table S2: Details of Surgical Procedures and Techniques used in the included studies. Table S3: Quality Assessment of non-randomized studies. Tabe S4: Results of Physical Examinations. Table S5: Pooled Incidence of Complications Following ACW-HTO with Revision ACLR. Table S6: Individual Study Complication Data for ACW-HTO with Revision ACLR.

## Abbreviations

The following abbreviations are used in this manuscript:

ACLR	Anterior Cruciate Ligament Reconstruction
PTS	Posterior Tibial Slope
ACW-HTO	Anterior Closed Wedge High Tibial Osteotomy
PROs	Patient-Reported Outcomes
IKDC	International Knee Documentation Committee
VAS	Visual Analogue Scale
ATT	Anterior Tibial Translation
RTS	Return to Sports
CDI	Caton–Deschamps Index
ISI	Insall–Salvati Index
SMD	Standardized Mean Difference
MD	Mean Difference
ROM	Range of Motion
CI	Confidence Interval
MINORS	Methodological Index for Non-Randomized Studies
WOSCC	Web of Science Core Collection
PRISMA	Preferred Reporting Items for Systematic Reviews and Meta-Analyses
AMSTAR	Assessing the Methodological Quality of Systematic Reviews
