# Supplementary material for: A Research Hotspot-Guided Meta-Analysis of Anterior Closing-Wedge High Tibial Osteotomy in Revision Anterior Cruciate Ligament Reconstruction
Source: Bioengineering (Basel). 2026 Mar 12;13(3):327. doi: 10.3390/bioengineering13030327 (PMC13024408; doi:10.3390/bioengineering13030327)
Supplement: Supplementary file 1 [file bioengineering-13-00327-s001.zip › Supplementary Files/PRISMA_2020_flow_diagram_new_SRs_v1.docx]

**Identification of studies via databases and registers**

Records identified from*:

Web of Science (n = 362)

Cochrane Library (n = 10)

PubMed (n = 222)

Embase (n = 259)

Records removed *before screening*:

Duplicate records removed (n = 333)

**Identification**

Records excluded** (n = 500)

Not related themes (n = 458)

Review articles (n = 13)

Non-ACL combined osteotomy

(n = 16)

Conference abstract (n = 4)

Laboratory or cadaveric studies (n = 9)

Records screened

(n = 520)

Reports not retrieved

(n = 0)

**Screening**

Reports sought for retrieval

(n = 20)

Reports Further excluded (n = 9):

Included primary and revision ACLR (n = 3)

Medial open-wedge osteotomy (n = 2)

Osteotomy for medial compartment osteoarthritis (n = 4)

Reports assessed for eligibility

(n = 20)

Studies included in meta-analysis

(n = 11)

**Included**

*Consider, if feasible to do so, reporting the number of records identified from each database or register searched (rather than the total number across all databases/registers).

**If automation tools were used, indicate how many records were excluded by a human and how many were excluded by automation tools.

Source: Page MJ, et al. BMJ 2021;372:n71. doi: 10.1136/bmj.n71.

This work is licensed under CC BY 4.0. To view a copy of this license, visit <https://creativecommons.org/licenses/by/4.0/>
