# Supplementary material for: A Research Hotspot-Guided Meta-Analysis of Anterior Closing-Wedge High Tibial Osteotomy in Revision Anterior Cruciate Ligament Reconstruction
Source: Bioengineering (Basel). 2026 Mar 12;13(3):327. doi: 10.3390/bioengineering13030327 (PMC13024408; doi:10.3390/bioengineering13030327)
Supplement: Supplementary file 1 [file bioengineering-13-00327-s001.zip › Supplementary Files/Supplementary file 1 (search strategy).docx]

**1.The Search strategy of Meta-analysis**

Search strategy in PubMed (May 31th, 2025)

| Search | Query | Results |
| --- | --- | --- |
| 4 | #1 AND #2 AND #3 | 222 |
| 3 | Sagittal tibial osteotomy [Title/Abstract] OR deviation osteotomy [Title/Abstract] OR slope reduction tibial osteotomy [Title/Abstract] tibial slope [Title/Abstract] | 2371 |
| 2 | Revision[Title/Abstract]OR revise[Title/Abstract] OR re-rupture [Title/Abstract] OR failure [Title/Abstract] | 3585 |
| 1 | anterior cruciate ligament reconstruction [Title/Abstract] OR ACLR[Title/Abstract] OR ACL reconstruction [Title/Abstract] | 1988 |

Search strategy in Web of Science (May 31th, 2025)

| Search | Query | Results |
| --- | --- | --- |
| #4 | #1 AND (#2 OR #3) | 362 |
| #3 | TS=(sagittal tibial osteotomy OR deviation osteotomy OR slope reduction tibial osteotomy OR tibial slope) | 789 |
| #2 | TS=(revision OR revise OR re-rupture OR failure) | 1231 |
| #1 | T=(anterior cruciate ligament reconstruction OR ACLR OR ACL reconstruction ) | 963 |

Search strategy in Embase (May 31th, 2025)

| No. | Query | Results | Date |
| --- | --- | --- | --- |
| #5 | #1 AND #2 AND #3 | 259 | 31-May-25 |
| #3 | 'saqital tibial osteotomy' :ti,ab,kw OR 'deviation osteotomy' :ti,ab,kw OR 'slope reduction tibial osteotomy ':ti,ab,kw OR 'tibial slope':ti,ab,kw | 954 | 31-May-25 |
| #2 | 'revision':ti,ab,kw OR 'revise':ti,ab,kw OR 're rupture':ti,ab,kw OR 'failure':ti,ab,kw | 1239 | 31-May-25 |
| #1 | 'anterior cruciate ligament reconstruction':ti,ab,kw OR 'aclr':ti,ab,kw OR 'acl reconstruction':ti,ab,kw | 1698 | 31-May-25 |

Search strategy in Cochrane Library (May 31th, 2025)

| Search | Query | Results |
| --- | --- | --- |
| #4 | #1 AND #2 AND #3 | 10 |
| #3 | '(anterior cruciate ligament recostruction ' OR ‘ACL reconstruction ' OR 'ACLR' ):ti,ab,kw | 507 |
| #2 | 'revision’ OR 'revise' OR 're-rupture'):ti,ab,kw | 1534 |
| #1 | 'Sagittal tibial osteotomy' OR 'deviation osteotomy' OR 'slope reduction tibial osteotomy' OR 'tibial slope'):ti,ab,kw | 923 |
