# Supplementary material for: A Research Hotspot-Guided Meta-Analysis of Anterior Closing-Wedge High Tibial Osteotomy in Revision Anterior Cruciate Ligament Reconstruction
Source: Bioengineering (Basel). 2026 Mar 12;13(3):327. doi: 10.3390/bioengineering13030327 (PMC13024408; doi:10.3390/bioengineering13030327)
Supplement: Supplementary file 1 [file bioengineering-13-00327-s001.zip › Supplementary Files/Supplementary file 2 (Sfigures).docx]

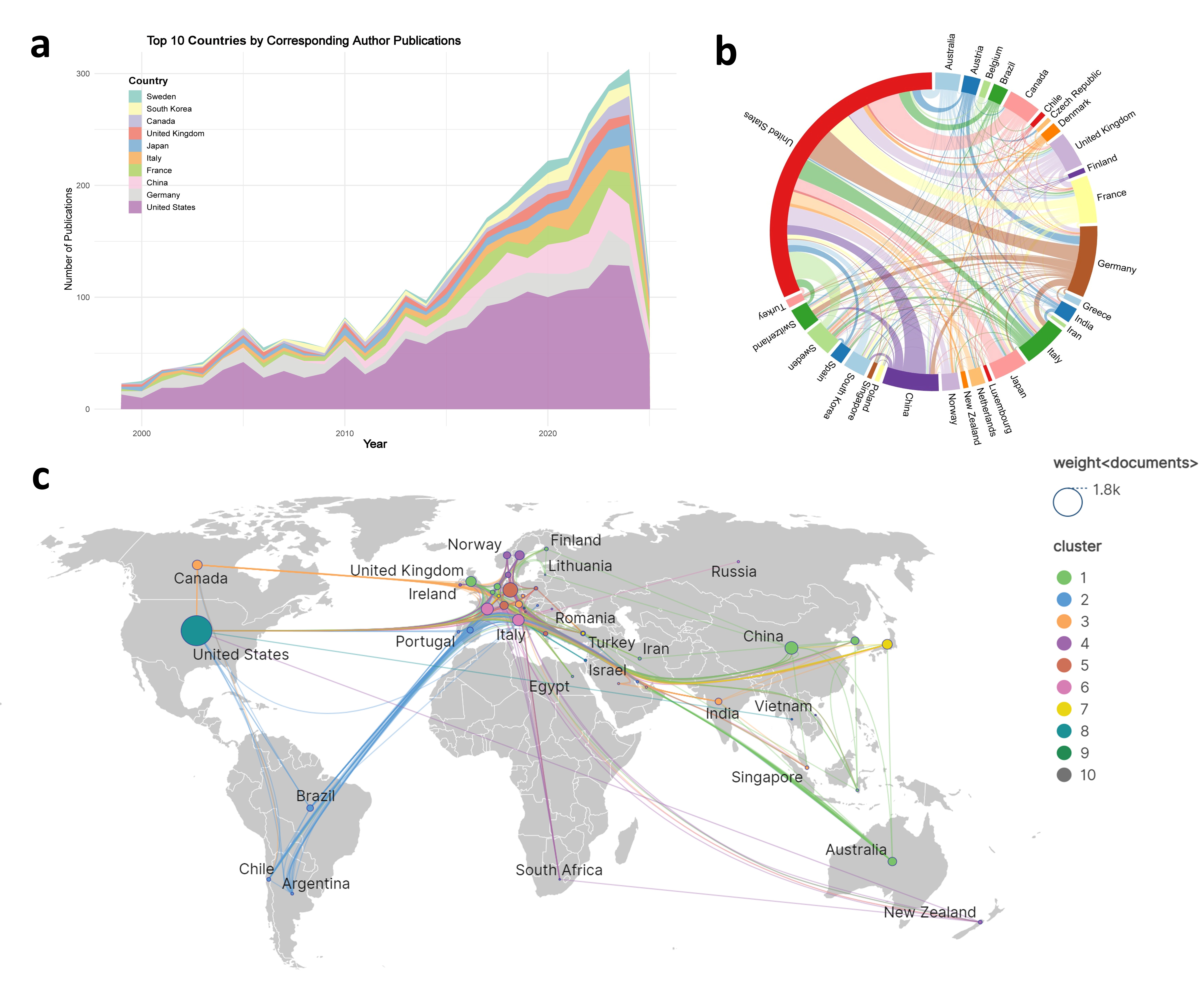


**Figure S1.** Productivity and Collaboration Among Nations in ACLR Revision Surgery Research. (a) Temporal distribution of output by high-producing countries. The countries are ordered and stacked from top to bottom according to their total publication volume. (b) Chord diagram illustrating collaborative relationships among the top 30 most productive countries. The width of the arcs represents the extent of co-authorship between countries. (c) Geographic Distribution Map of Publication by Country in ACLR Revision Surgery Research. The colors representing countries/regions have no specific meaning; only the thickness of the lines between them is significant, indicating the frequency of collaboration between different countries. The thickness of the lines corresponds to the values on their respective axes.


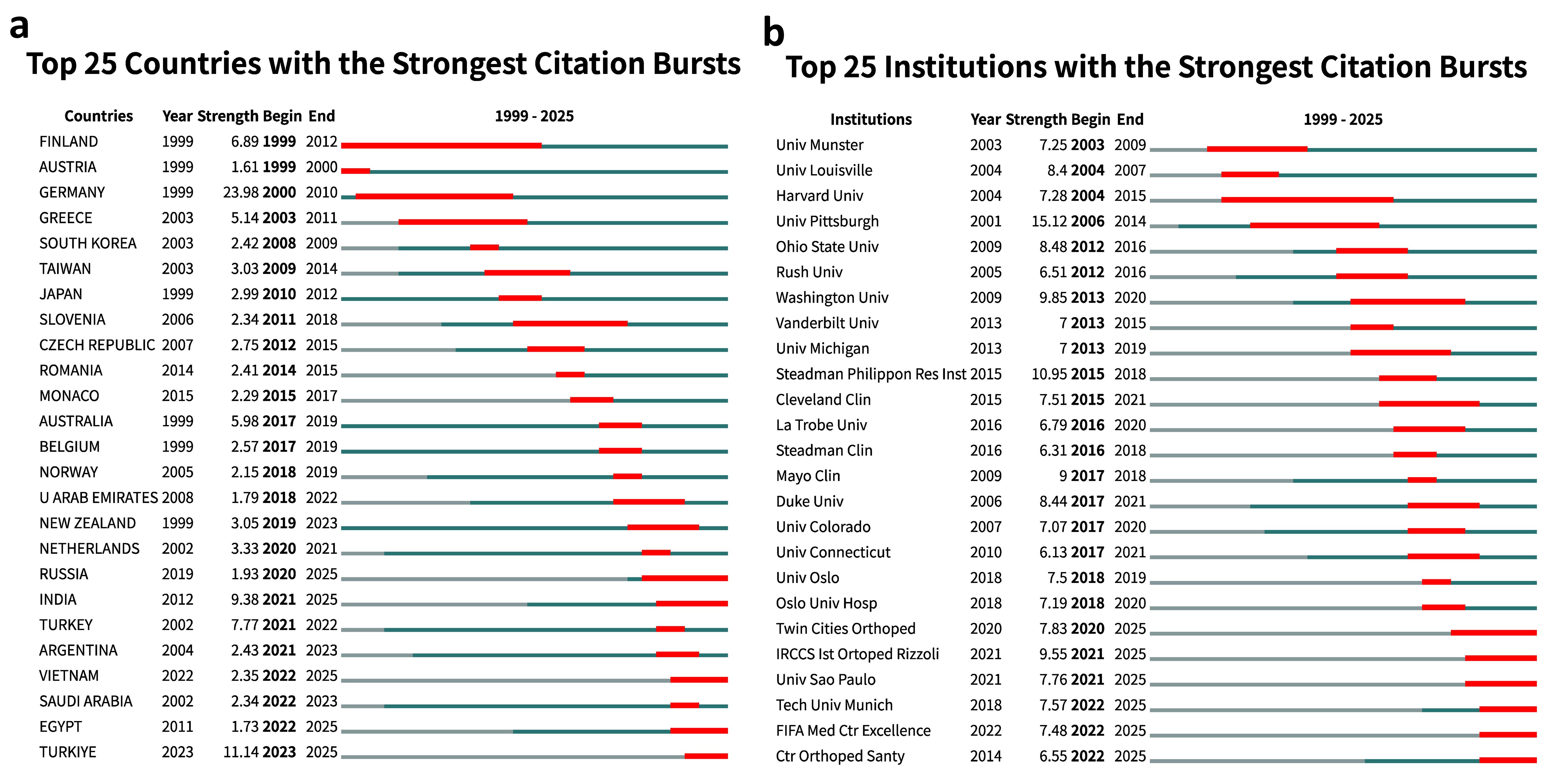


**Figure S2:** Institutional distribution, productivity, and collaboration in ACLR Revision Surgery Research. (A) Proportion of publications by institution type. (B) Stacked area chart of the top 10 most productive institutions from 1999 to 2023, ordered and stacked by total publication volume. (C) Collaboration network map among major institutions generated by VOSviewer. (D) Top 20 institutions with the strongest citation bursts between 1999 and 2025, with red segments indicating periods of high citation intensity.


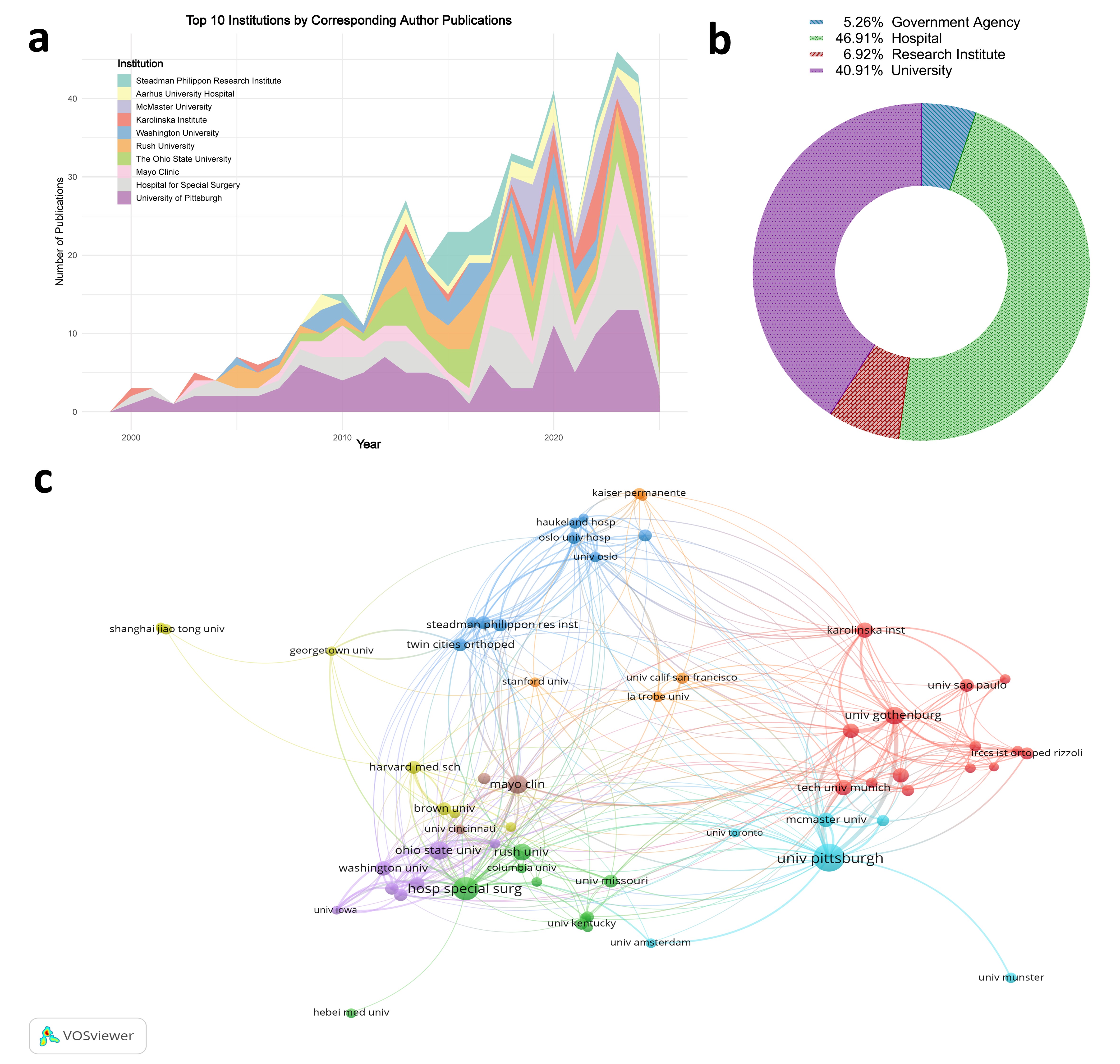


**Figure S3.** Burst Citation Analysis of Countries and Institutions. (a) Top 25 Countries with the Strongest Citation Bursts. (b) Top 25 Institutions with the Strongest Citation Bursts. The red bars in the chart represent the periods of citation bursts for each country and institution. The year value listed next to each institution or country indicates the specific time period during which the strongest burst in citations occurred.


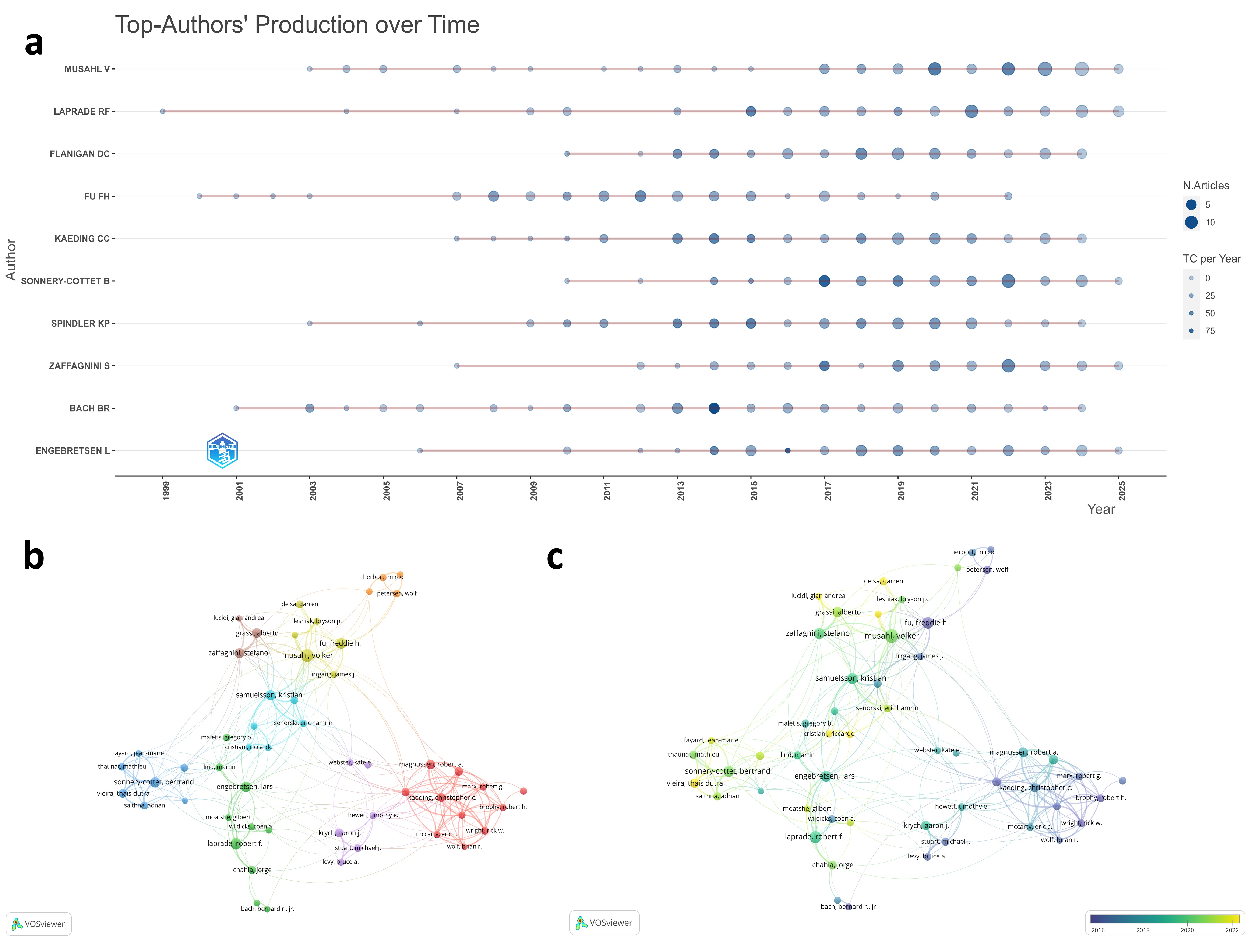


**Figure S4.** Author productivity, collaboration, and citation dynamics in ACLR revision surgery research. (a) Temporal distribution of publications by the top 10 authors. Each circle represents the number of articles published in a given year, with circle size proportional to the number of articles and color depth reflecting the average citations per article per year. (b) Author collaboration network generated by VOSviewer (n ≥ 20). (c) Author collaboration clustering and temporal distribution analysis. Each node represents an author, node size indicates publication volume, node color denotes cluster membership, and the thickness of connecting lines reflects the strength of co-authorship links.


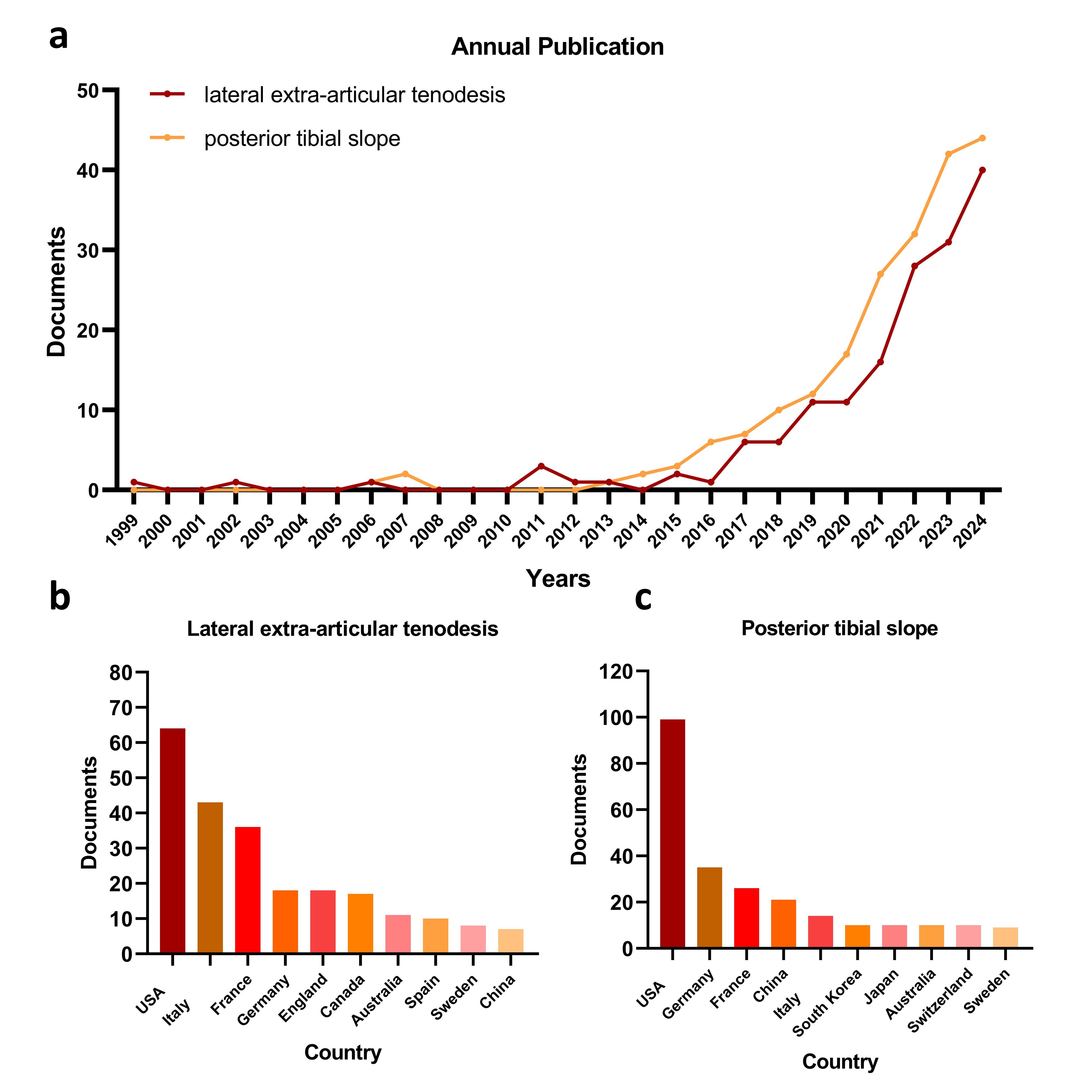


**Figure S5.** Research sub-directions in ACLR revision surgery: Publication trends and country analysis. (a) Annual publication trends for two research sub-directions, lateral extra-articular tenodesis (red line) and posterior tibial slope (orange line). (b) Country distribution of publications on lateral extra-articular tenodesis. (c) Country distribution of publications on posterior tibial slope.


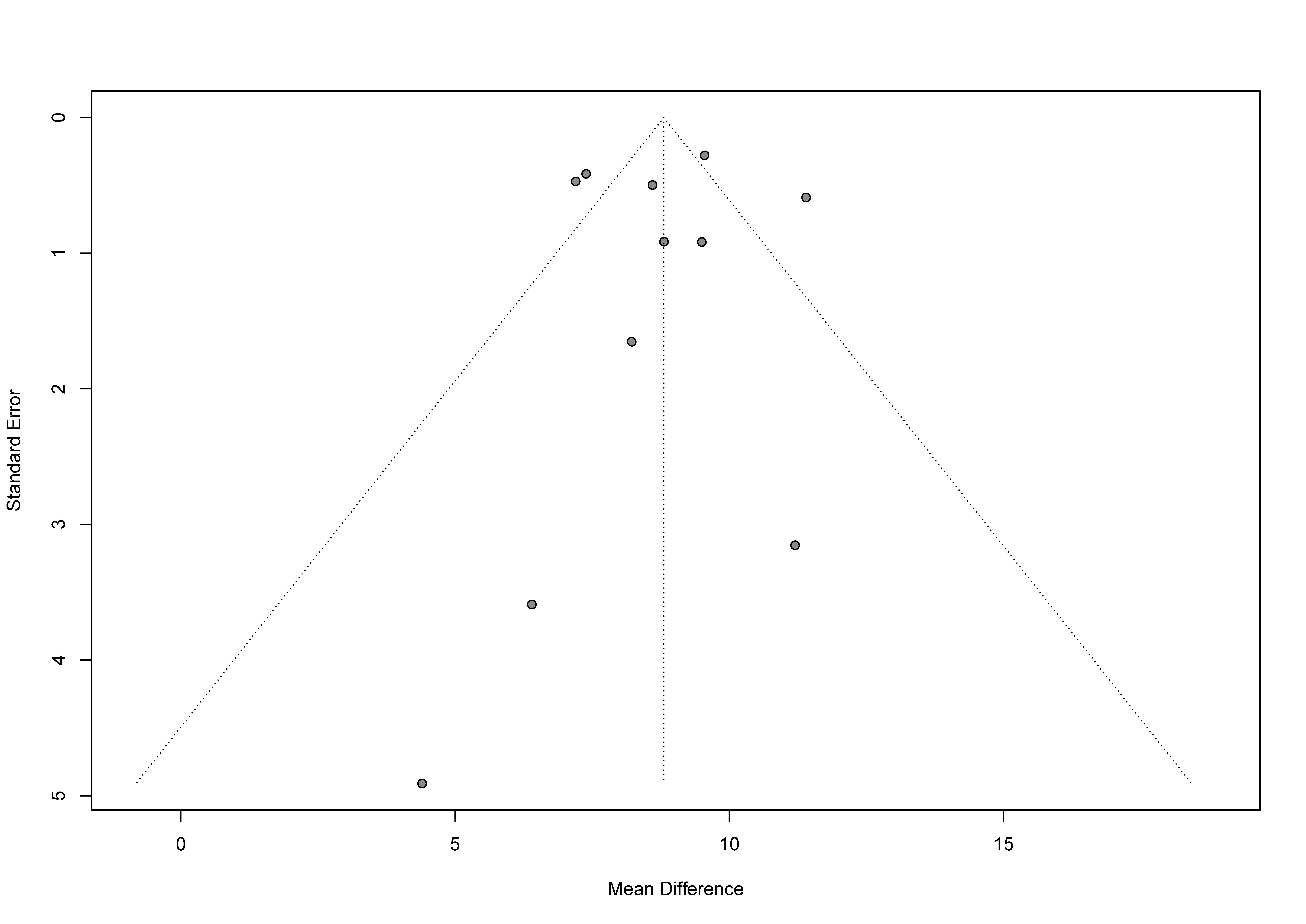


**Figure S6:** publication bias for the PTS outcome.
