# Supplementary material for: A Research Hotspot-Guided Meta-Analysis of Anterior Closing-Wedge High Tibial Osteotomy in Revision Anterior Cruciate Ligament Reconstruction
Source: Bioengineering (Basel). 2026 Mar 12;13(3):327. doi: 10.3390/bioengineering13030327 (PMC13024408; doi:10.3390/bioengineering13030327)
Supplement: Supplementary file 1 [file bioengineering-13-00327-s001.zip › Supplementary Files/Supplementary file 3 (sensitivity analysis results).docx]

**Leave one out meta-analysis results**

**ATT**

**
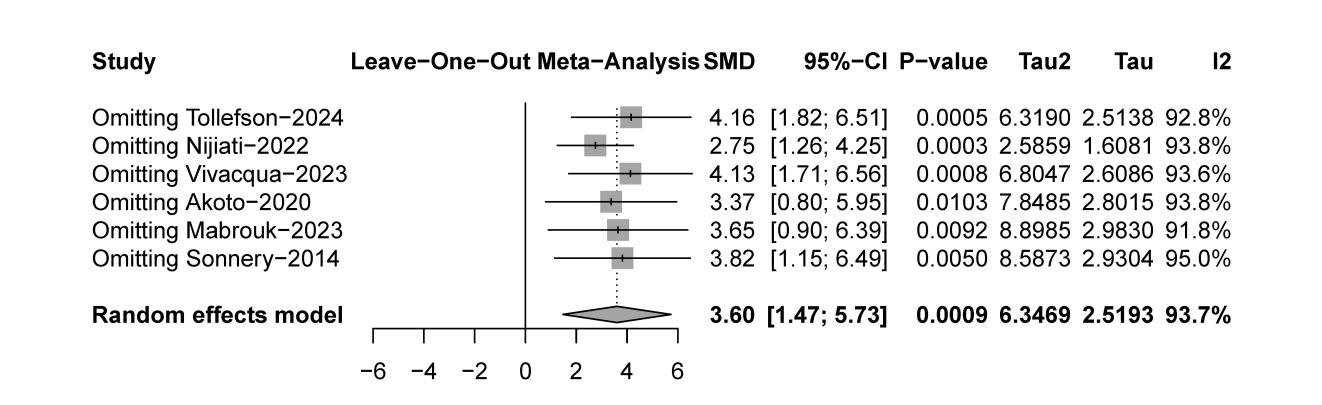
**

**IKDC**

**
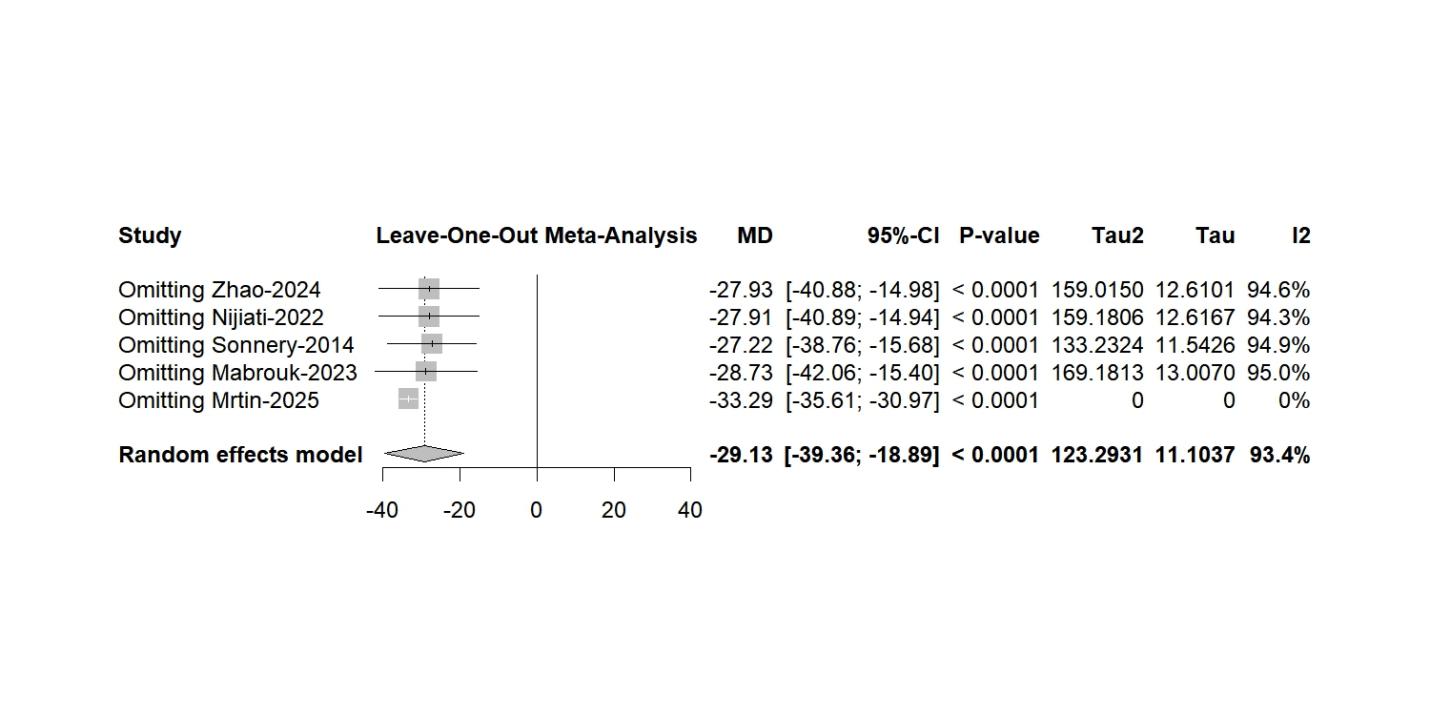
**

**Lysholm**

**
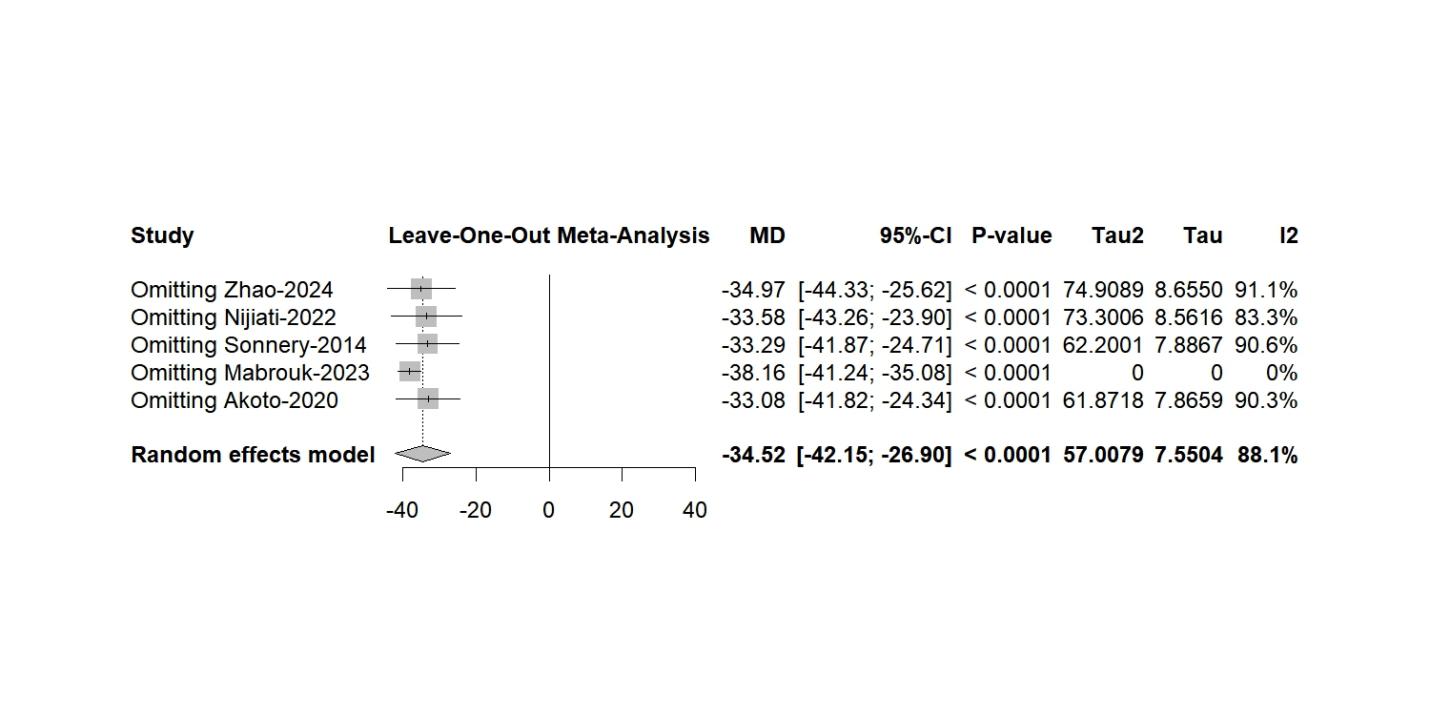
**

**Patella height indices**

**
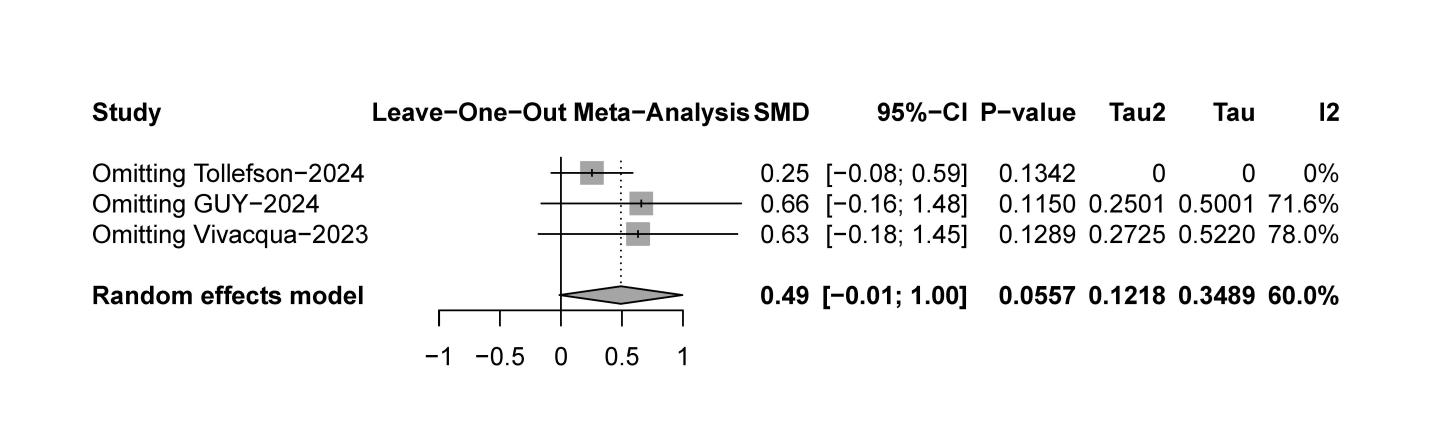
**

**PTS**

**
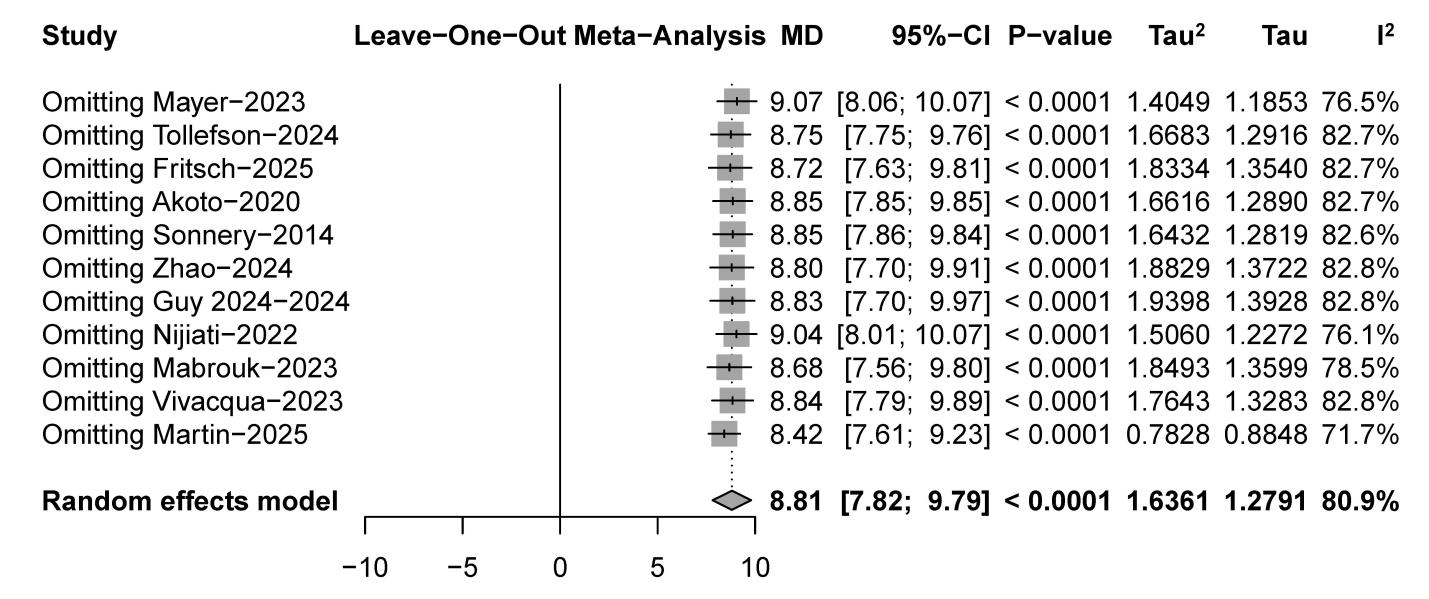
**

**Tegner**

**
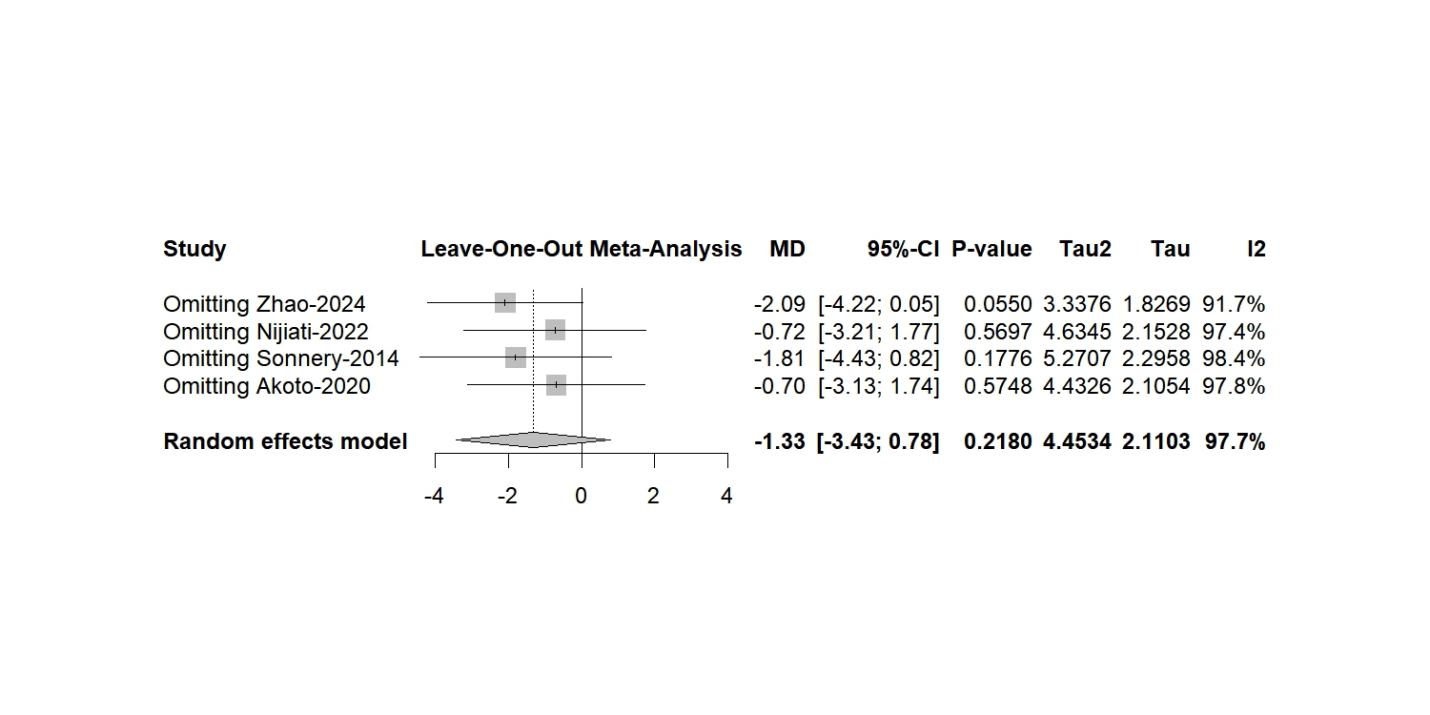
**

**Subgroup meta-analysis of PTS**

**
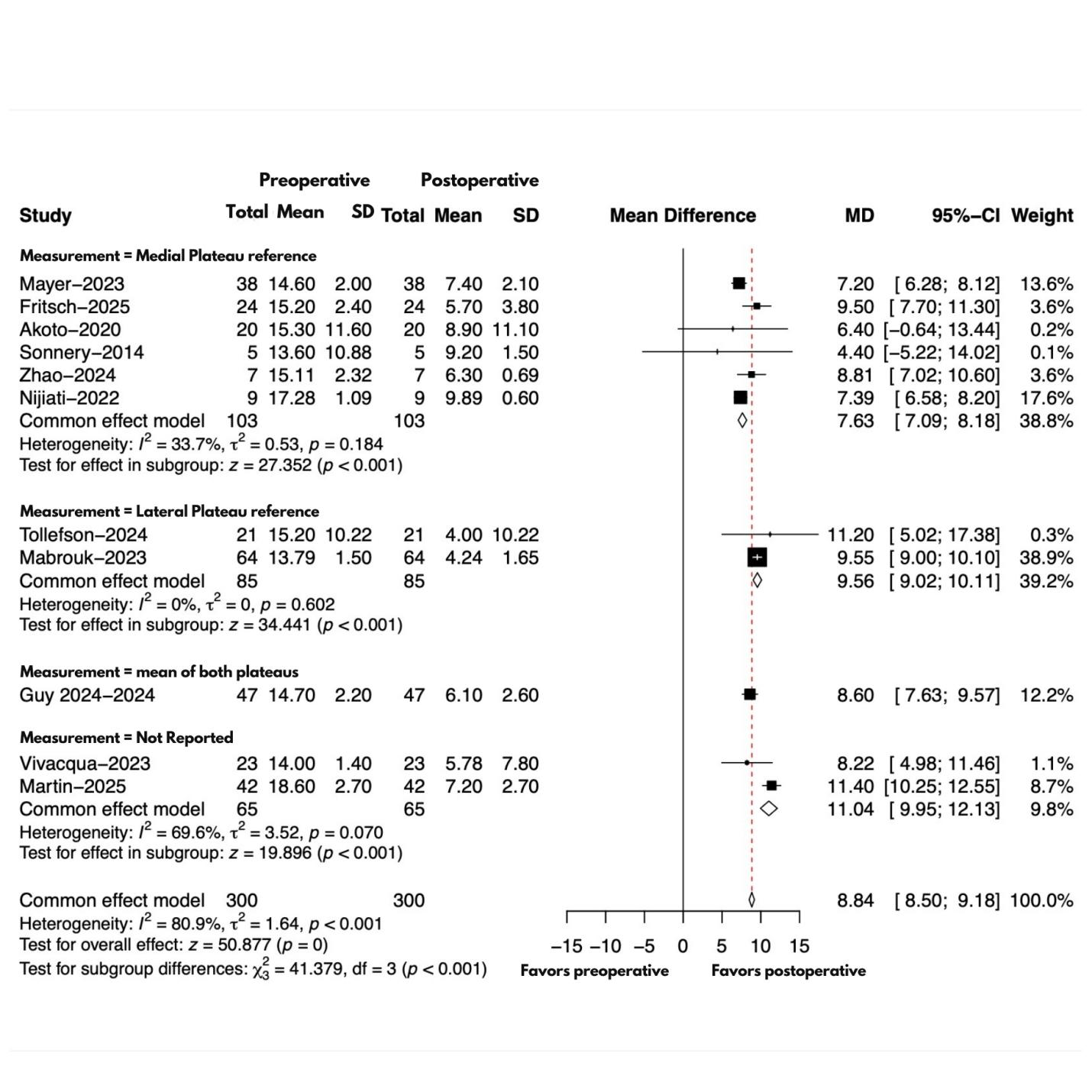
**
