# Supplementary material for: A Research Hotspot-Guided Meta-Analysis of Anterior Closing-Wedge High Tibial Osteotomy in Revision Anterior Cruciate Ligament Reconstruction
Source: Bioengineering (Basel). 2026 Mar 12;13(3):327. doi: 10.3390/bioengineering13030327 (PMC13024408; doi:10.3390/bioengineering13030327)
Supplement: Supplementary file 1 [file bioengineering-13-00327-s001.zip › Supplementary Files/Table S1.docx]

**Table S1:** Basic characteristics of included studies.

| First author-Year | Country | Study Design-LOE | Sample size, initial  cohort/final cohort | Follow-up(months) | Sex (Male/Female) | Age (years) | Procedure | Fixation Methods | Indication | Outcomes measured |
| --- | --- | --- | --- | --- | --- | --- | --- | --- | --- | --- |
| Fritsch-2025 | Germany | RCS IV | 26/24 | 34.3 months | 18/6 | Mean 27 ± 8 | Infra-tuberosity ACW-HTO | Interference screw, monocortical screw | previous ACLR failure, PTS  >12° | VAS, IKDC, PTS,  KOOS, Lysholm, TAS, ATT, Lachman |
| Martin-2025 | Japan | PCS II | 42/42 | 60 months | 25/17 | Mean 25.1±6.7 | Trans-tuberosity ACW-HTO | staples  or locking plate | previous ACLR failure, PTS  >15° | ACL-RSI, IKDC, KOOS, return to sports  and graft failure |
| Guy-2024 | France | RCS IV | 47/47 | 35 months | 32/15 | 28.9±9.2  range (17-58) | Trans-tuberosity ACW-HTO | Two staples | 1 prior ACLR failure and a PTS  >12° | PTS, CDI, ISI, patella height |
| Tollefson-2024 | USA | RCS IV | 20/20 | 6 months | 6/14 | 26.9  range (18-36) | Supra-tuberosity ACW-HTO | Three staples | 1 prior ACLR failure and a PTS  >12° | PTS, ATT, CDI |
| Mabrouk-2023 | France | RCS IV | 64/64 | 30 months | 53/11 | 29.60±6.31 | Supra-tuberosity ACW-HTO | Medial locked plate  or  2 staples | Third revision ACLR, PTS  >12° | PTS, IKDC, Lysholm, ATT,  pivot-shift, ROM |
| Vivacqua-2023 | USA | RCS IV | 23/23 | 26.7  months | 12/11 | Mean 28.7± 9.8 | Supra-tuberosity (19) and trans-tuberosity (4) ACW-HTO | Staples or plates | prior ACLR failure and a PTS  >12° | PTS, CDI, IKDC, ATT |
| Mayer-2023 | Germany | RCS IV | 39/38 | NR | 24/14 | 31.6±8.7  range (17-51) | Infra-tuberosity ACW-HTO | Anteromedial plate  fixation | >1 previous ACLR failure | PTS, MPTA, ROM |
| Nijiati-2022 | China | RCS IV | 9/9 | 25  months | 8/1 | Range (21-42) | Infra-tuberosity ACW-HTO | Adjustable button AND interference screw | previous ACLR failure, PTS  >17° | PTS, ATT, IKDC, Lysholm, Tegner |
| Akoto-2020 | Germany | RCS IV | 22/20 | 30 months | 14/6 | 27.8±8.6  Range (18-49) | Trans-tuberosity ACW-HTO | Three screws  through the  tubercle | Previous ACLR failure, PTS  >12° | PTS, IKDC, Lysholm, Tegner,  ATT, pivot-shift, Lachman, |
| Sonnery-2014 | France | RCS IV | 5/5 | 31.6 months | 4/1 | 24  Range (16-40) | Trans-tuberosity ACW-HTO | Screws through  tibial tubercle and  staples | previous ACLR failure, PTS  >12° | PTS, IKDC, Lysholm, ATT,  pivot-shift, TAS, VAS |
| Zhao 2024 | China | RCS IV | 7/7 | 28.1 months | 2/5 | 34  Range (5-58) | Infra-tuberosity ACW-HTO | Locking plate AND osteotomy buttress screw | previous ACLR failure, PTS  >12° | IKDC, Lysholm, TAS, PTS |

Abbreviation:

RCS: Retrospective case aeries, PCS: Prospective case series, LOE: Level of evidence, ACW-HTO: Anterior Closing wedge high tibial osteotomy, ACLR: Anterior cruciate ligament, PTS: Posterior tibial slope, IKDC: International knee documentation committee, TAS: Tegner activity score, PTS: Posterior tibial slope, ATT, Anterior tibial translation, ROM: Range of motion, MPTA: Medial proximal tibial angle, CDI: Caton-Deschamps index, ISI: Insall-Salvati index, RTS: Return to sport, KOOS: Knee injury and osteoarthritis outcome Score, VAS: Visual analogue score.
