# Supplementary material for: A Research Hotspot-Guided Meta-Analysis of Anterior Closing-Wedge High Tibial Osteotomy in Revision Anterior Cruciate Ligament Reconstruction
Source: Bioengineering (Basel). 2026 Mar 12;13(3):327. doi: 10.3390/bioengineering13030327 (PMC13024408; doi:10.3390/bioengineering13030327)
Supplement: Supplementary file 1 [file bioengineering-13-00327-s001.zip › Supplementary Files/Table S2.docx]

**Table S2 :** Details of Surgical Procedures and Techniques used in the included studies ^a^

| **First Author (Year)** | **ACLR Revision n(N%)** | **Stage**  **Procedure ACLR** | **Infratuberosity**  **Approach** | **Transtuberosity**  **Approach** | **Supratuberosity**  **Approach** | **Method**  **of measuring PTS** | **Concomitant approach** | **Meniscal Procedure, n** | **QT Graft** | **HT**  **Graft** | **BPTB**  **Graft** | **Allograft** | **Osteotomy surgical technique** |
| --- | --- | --- | --- | --- | --- | --- | --- | --- | --- | --- | --- | --- | --- |
| Sonnery-Cottet (2014) | 5 (100) | Single  Stage | 0 (0) | 5 (100) | 0 (0) | Medial tibial plateau | None | LM partial resection: 1  LM repair: 1 | 4 | 0 | 1 | 0 | ACW-HTO with detachment of ATT and  patellar tendon |
| Akoto (2020) | 20 (100) | Two Stage | 0 (0) | 20 (100) | 0 (0) | Medial tibial plateau | LET: 20 (100) | MM repair: 8  MM partial resection: 3  MM total resection: 1 | 12 | 7 | 1 | 0 | ACW-HTO with detachment of ATT and  patellar tendon |
| Nijiati 2022 | 9(100) | Single  Stage | 9(100) | 0(0) | 0 (0) | Medial tibial plateau | NR | MM partial resection: 1  LM repair:1  LM partial resection: 1 | 0 | 9 | 0 | 0 | ACW-HTO without detachment of  ATT and patellar tendon |
| Fritsch 2025 | 24(100 | Two Stage | 24(100) | 0(0) | 0(0) | Medial tibial plateau | LET: 16 | MM partial resection:1  MM repair:6  LM partial resection: 2  LM repair:6 | 16 | 5 | 0 | 3 | ACW-HTO  without detachment of  ATT and patellar tendon |
| Martin 2025 | 42(100) | Two Stage | 0(0) | 42(100) | 0(0) | NR | NR | NR | NR | NR | NR |  | ACW-HTO with detachment of ATT and  patellar tendon |
| Guy 2024 | 47(100) | Single Stage | 0(0) | 47(100) | 0(0) | Mean of both plateaus | NR | NR | 0 | 0 | 0 | 47 | ACW-HTO  without detachment of  ATT and patellar tendon |
| Tollefson 2024 | 20(100) | Two Stage | 0(0) | 0(0) | 20(200) | Lateral tibial plateau | NR | NR | NR | NR | NR | NR | ACW-HTO  without detachment of  ATT and patellar tendon |
| Mabrouk 2023 | 64(100) | Single  Stage | 0(0) | 0(0) | 64(100) | Lateral tibial plateau | LET: 46 | MM partial resection: 22  MM total resection: 5  LM partial resection: 5  LM total  resection: 2  Meniscal repair:54 | 0 | 19 | 22 | 18 | ACW-HTO  without detachment of  ATT and patellar tendon |
| Vivacqua 2023 | 23 | Single Stage:16  Two Stage:7 | 0 | 4(17.4) | 19(82.6) | NR | LET:7 | MM resection:5  LM resection:4  MM repair:4  LM repair:5  Medial MAT:2 | 9 | 0 | 5 | 9 | ACW-HTO with or without detachment of  ATT and patellar tendon |
| Mayer 2023 | 38(100) | Single  Stage:18  Two Stage:20 | 38(100) | 0(0) | 0(0) | Medial tibial plateau | LET:18 | NR | 10 | 6 | 0 | 2 | ACW-HTO  without detachment of  ATT and patellar tendon |
| Zhao 2024 | 7(100) | Single Stage | 7(100) | 0(0) | 0(0) | Medial tibial plateau | NR | NR | 1 | 5 | 1 | 0 | ACW-HTO  without detachment of  ATT and patellar tendon |

a: Data are expressed as n (%) unless otherwise specified. ACLR, anterior cruciate ligament reconstruction; ACW-HTO— anterior closed-wedge high tibial osteotomy; QT—quadriceps tendon; HT—hamstring tendon; BPTB—bone-patellar tendon-bone; Single stage: slope-reducing tibial osteotomy combined with ACLR; Two stage: slope-reducing tibial osteotomy was performed first, and then revision ACLR; LET, lateral extra-articular tenodesis; MM, medial meniscus; LM, lateral meniscus; ATT—anterior tibial tuberosity; NR, not reported.
