# Supplementary material for: A Research Hotspot-Guided Meta-Analysis of Anterior Closing-Wedge High Tibial Osteotomy in Revision Anterior Cruciate Ligament Reconstruction
Source: Bioengineering (Basel). 2026 Mar 12;13(3):327. doi: 10.3390/bioengineering13030327 (PMC13024408; doi:10.3390/bioengineering13030327)
Supplement: Supplementary file 1 [file bioengineering-13-00327-s001.zip › Supplementary Files/Table S3.docx]

**Table S3.** Quality Assessment of non-randomized studies

| Study  (author-year) | Methodological items for non-randomized studies | | | | | | | | |
| --- | --- | --- | --- | --- | --- | --- | --- | --- | --- |
|  | Aim | Consecutive Patients | Prospective Data | Endpoints | Unbiased Assessment | Adequate Follow-up | <5% Loss to Follow-up | Sample Size Calculation | Total Score |
| Akoto-2020 | 2 | 2 | 0 | 2 | 0 | 2 | 2 | 0 | 10 |
| Fritsch-2025 | 2 | 2 | 0 | 2 | 0 | 2 | 2 | 0 | 10 |
| Guy -2024 | 2 | 2 | 0 | 2 | 0 | 2 | 2 | 0 | 10 |
| Martin-2025 | 2 | 2 | 1 | 2 | 0 | 0 | 2 | 0 | 9 |
| Nijiati-2022 | 2 | 2 | 0 | 2 | 0 | 2 | 2 | 0 | 10 |
| Mabrouk-2023 | 2 | 2 | 1 | 2 | 0 | 2 | 2 | 0 | 11 |
| Mayer-2023 | 2 | 2 | 0 | 2 | 0 | 0 | 2 | 0 | 8 |
| Sonnery-2014 | 2 | 2 | 0 | 2 | 0 | 2 | 2 | 0 | 10 |
| Vivacqua-2023 | 2 | 2 | 0 | 2 | 2 | 2 | 2 | 0 | 12 |
| Zhao-2024 | 2 | 2 | 0 | 2 | 2 | 2 | 2 | 0 | 12 |
| Tollefson-2024 | 2 | 2 | 0 | 2 | 0 | 0 | 2 | 0 | 8 |
