# Supplementary material for: A Research Hotspot-Guided Meta-Analysis of Anterior Closing-Wedge High Tibial Osteotomy in Revision Anterior Cruciate Ligament Reconstruction
Source: Bioengineering (Basel). 2026 Mar 12;13(3):327. doi: 10.3390/bioengineering13030327 (PMC13024408; doi:10.3390/bioengineering13030327)
Supplement: Supplementary file 1 [file bioengineering-13-00327-s001.zip › Supplementary Files/Table S4.docx]

**Tabe S4.** Results of Physical Examinations

| Outcomes | Author-Year | Grade | Pre-op | Post-op | P value | Follow-up |
| --- | --- | --- | --- | --- | --- | --- |
| Pivot shift | Mabrouk-2023 | 0/1/2/3 | 15/17/22/10 | 22/25/14/3 | NS | 60 months |
|  | Nijiati-202 | 0/1/2/3 | 6/2/1/0 | 9/0/0/0 | NS | 36 months |
|  | Akoto-2020 | 0/1/2/3 | 0/0/0/20 | 20/0/0/0 | P<0.001 | 30 months |
|  | Sonnery-Cotte,2014 | 0/1/2/3 | 0/1/3/1 | 4/1/0/0 | NS | 31 months |
| Lachman-test | Akoto-2020 | 1/2/3 | 0/11/9 | 1/0/0 | NS | 24 months |
|  | Fritsch-2025 | 1/2/3 | NR | 17/6/1 | NS | 24 months |
