# Supplementary material for: A Research Hotspot-Guided Meta-Analysis of Anterior Closing-Wedge High Tibial Osteotomy in Revision Anterior Cruciate Ligament Reconstruction
Source: Bioengineering (Basel). 2026 Mar 12;13(3):327. doi: 10.3390/bioengineering13030327 (PMC13024408; doi:10.3390/bioengineering13030327)
Supplement: Supplementary file 1 [file bioengineering-13-00327-s001.zip › Supplementary Files/Table S5-6.docx]

**Table S5.** Pooled Incidence of Complications Following ACW-HTO with Revision ACLR

| **Complication Type** | **Number of Events** | **Total Patients in Reporting Studies** | **Incidence Rate (%)** |
| --- | --- | --- | --- |
| **Symptomatic Hardware** | 25 | 125 | **20.0%** |
| **Recurvatum** | 35 | 211 | **16.6%** |
| **Graft Failure** | 10 | 189 | **5.3%** |
| **Infection** | 2 | 89 | **2.3%** |
| **Other Complications** | 8 | 132 | **6.1%** |

**Table S6.** Individual Study Complication Data for ACW-HTO with Revision ACLR

| **Author-Year** | **Total patients** | **Graft Failure** | **Infection** | **Symptomatic Hardware** | **Recurvatum** | **Others** | **Total Complications** |
| --- | --- | --- | --- | --- | --- | --- | --- |
| Akoto-2020 | 20 | 0 | NR | NR | 3 | 1 | 4 |
| Fritsch-2025 | 24 | 4 | NR | NR | 11 | NR | 15 |
| Martin-2025 | 42 | 0 | 1 | NR | 0 | 3 | 4 |
| Mabrouk-2023 | 64 | 3 | NR | 11 | 21 | NR | 35 |
| Vivacqua-2023 | 23 | 3 | NR | 6 | 0 | 4 | 13 |
| Mayer-2023 | 38 | NR | 1 | 8 | 0 | 0 | 9 |
| Nijiati-2022 | 9 | 0 | 0 | NR | NR | 0 | 0 |
| Zhao-2024 | 7 | 0 | NR | NR | NR | NR | 0 |
| Guy-2024 | 47 | NR | NR | NR | NR | NR | NR |
| Tollefson-2024 | 20 | NR | NR | NR | NR | NR | NR |
| Sonnery-2014 | 5 | NR | NR | NR | NR | NR | NR |

**Footnote:** ACW-HTO = Anterior Closing-Wedge High Tibial Osteotomy; ACLR = Anterior Cruciate Ligament Reconstruction; NR = Not Reported.
